# Supplementary material for: Safety, efficacy and total cost of point-of-care manufactured anti-CD19 CAR-T cell therapy in India: VELCART trial
Source: Mol Ther Oncol. 2025 Mar 25;33(2):200977. doi: 10.1016/j.omton.2025.200977 (PMC12005290; doi:10.1016/j.omton.2025.200977)
Supplement: Document S1. Figures S1S8, Tables S1–S5, and Supplemental Methods [file mmc1.pdf]

OMTON, Volume *33*

Supplemental information

Safety, efficacy and total cost of point-of-care manufactured anti-CD19 CAR-T cell therapy in India: VELCART trial

Hamenth Kumar Palani, Arun Kumar Arunachalam, Uday Kulkarni, Mohammed Yasar, Arvind Venkatraman, Swathy Palanikumar, Reeshma Nair Radhakrishnan, Majeela Solomon, Abirami Rajasekaran, Aniket Bankar, Phaneendra Venkateswara Rao Datari, Sushil Selvarajan, Anu Korula, Pradyot Dash, Dina Schneider, Louisa Wirthlin, Aby Abraham, Biju George, and Vikram Mathews

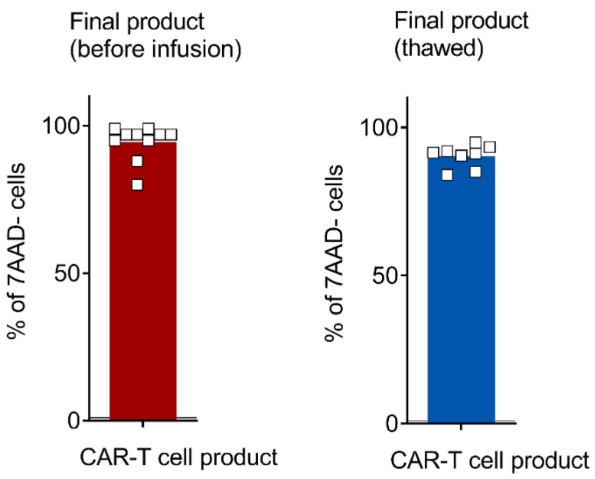

Figure S1. Viability analysis of cryopreserved CAR T-cell products thawed after six months. The viability was evaluated using 7AAD staining by flow cytometry after overnight resting with a TexMACS medium.

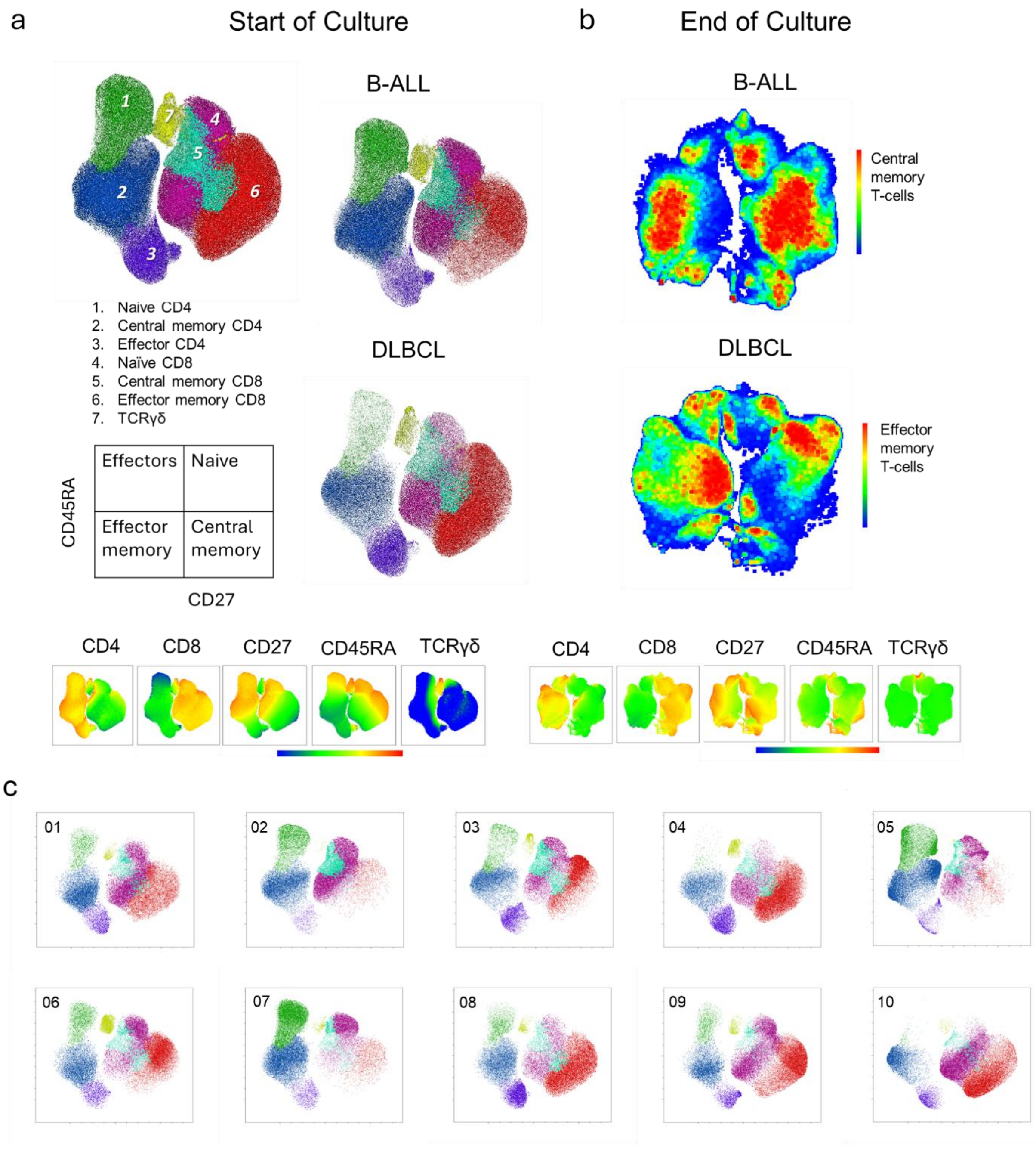

Figure S2. Identification and clustering of starting T-cells based on marker expression profiles. a) Clustering analysis of starting T-cells before culture. T-cells were identified with CD3 after sequentially excluding doublets, dead cells, CD45 negative events, B-cells, and monocytes. Unsupervised clustering via FLOWSOM, after dimensionality reduction through UMAP using FlowJo™ software. Multigraph color mapping demonstrates the expression of different markers.

b) Two-dimensional UMAP (uniform manifold approximation and projection) plot and the corresponding heatmap statistics showing the increased density of CD27 negative effector T-cells (CD4 & CD8) in the DLBCL samples and CD27 positive central memory T-cells (CD4 & CD8) in the B-ALL samples. c) The distribution of all clusters in the starting T-cells of all samples in the cohort.

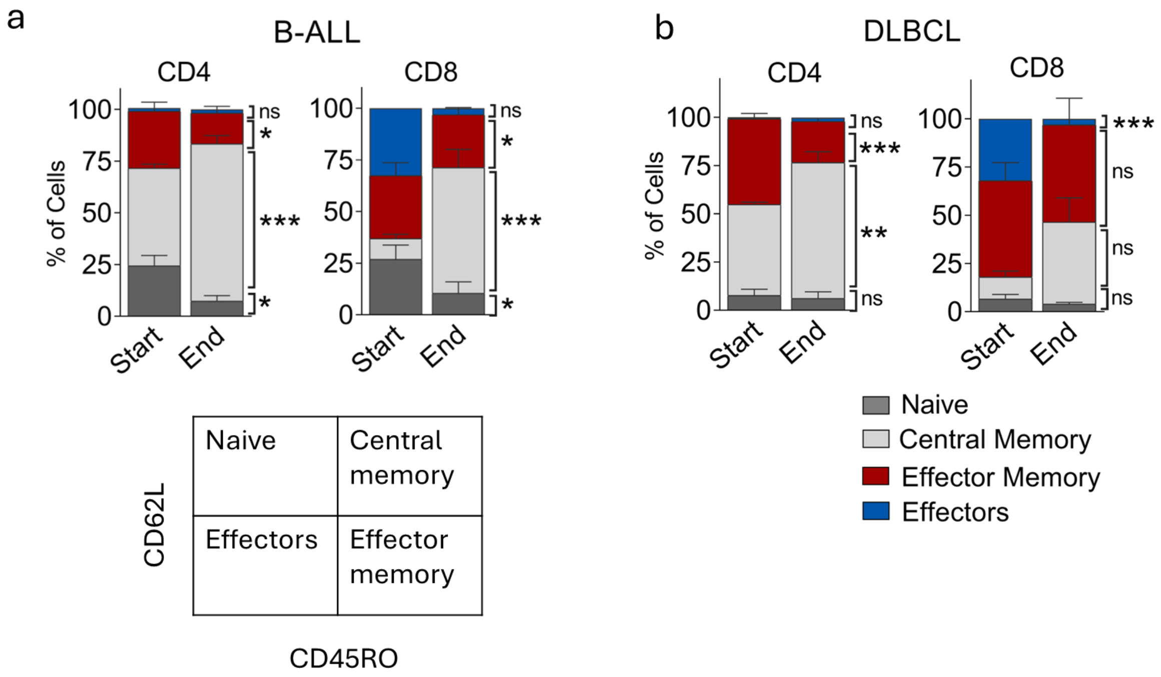

Figure S3. T-cell subset analysis of final CAR T-cell products comparing their starting T-cells. a) Percentage of cells in CD4 and CD8 subsets comparing the starting T-cell culture and final CAR T-cell products in B-ALL and DLBCL cohorts (b).

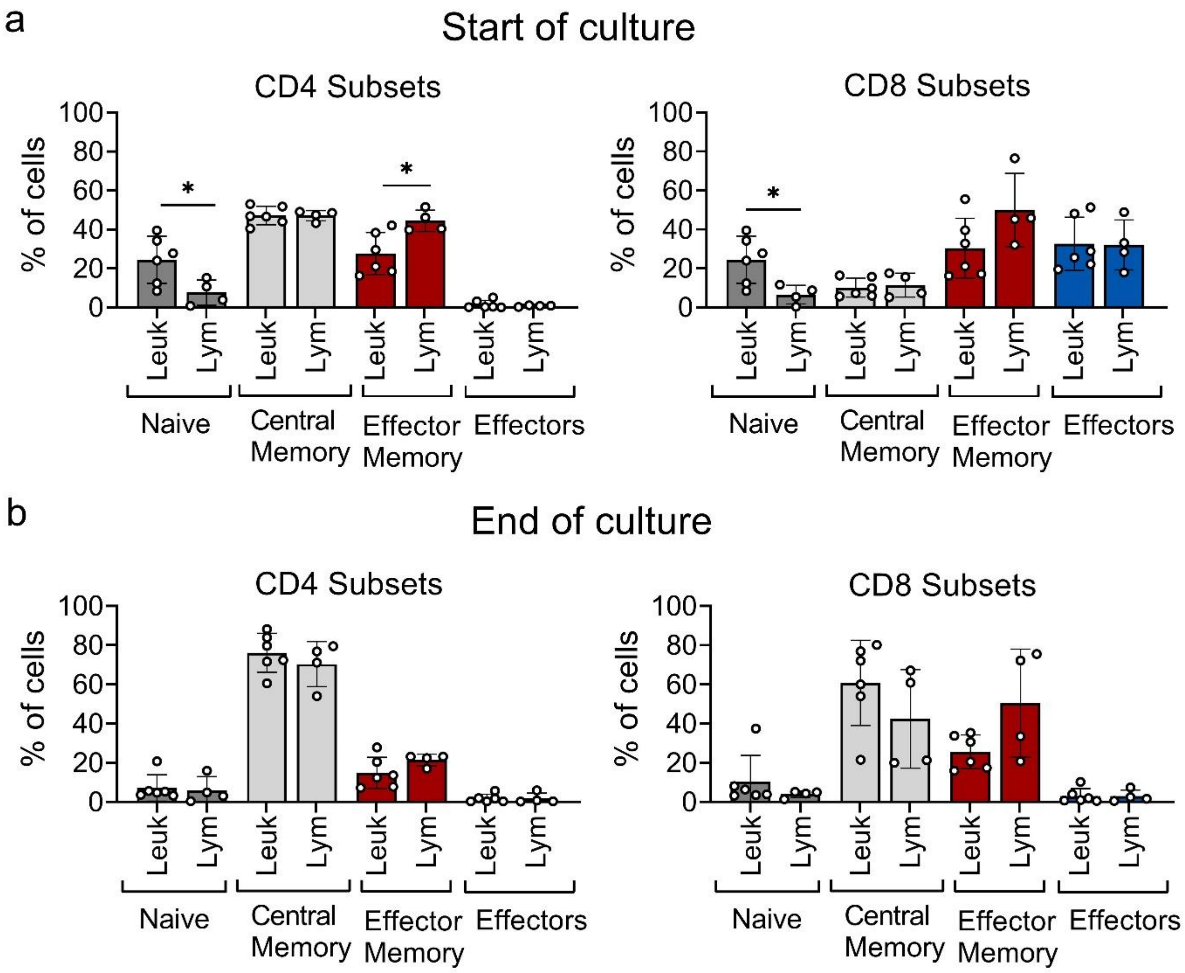

Figure S4. T-cell subset analysis of CAR T-cell products comparing leukemia and lymphoma. a) Immunophenotypic characterization of T-cell subsets comparing leukemia (B-ALL) and lymphoma (DLBCL) samples at the start of culture. b) Comparison of T-cell subsets at the end of culture. Each dot represents each sample n=8.

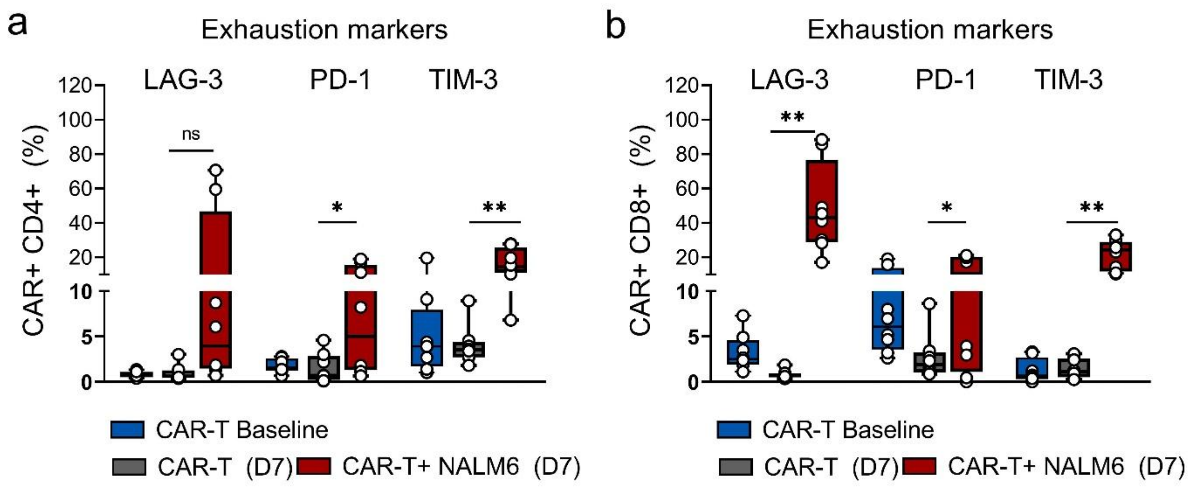

Figure S5. Expression of exhaustion marker on CAR T-cell restimulated with target cells. The expression of exhaustion makers of CAR T-cells after antigen-specific stimulation was done by an *in vitro* restimulation assay, where CAR T-cells were co-cultured with an equal number of target cells (NALM6) with intermittent stimulation for 6 days. a) Percentage of LAG-3, PD-1 and TIM-3 among CAR+ CD4+ cells. b) Percentage of markers among CAR+ CD8+ cells. Each dot represents each sample n=8.

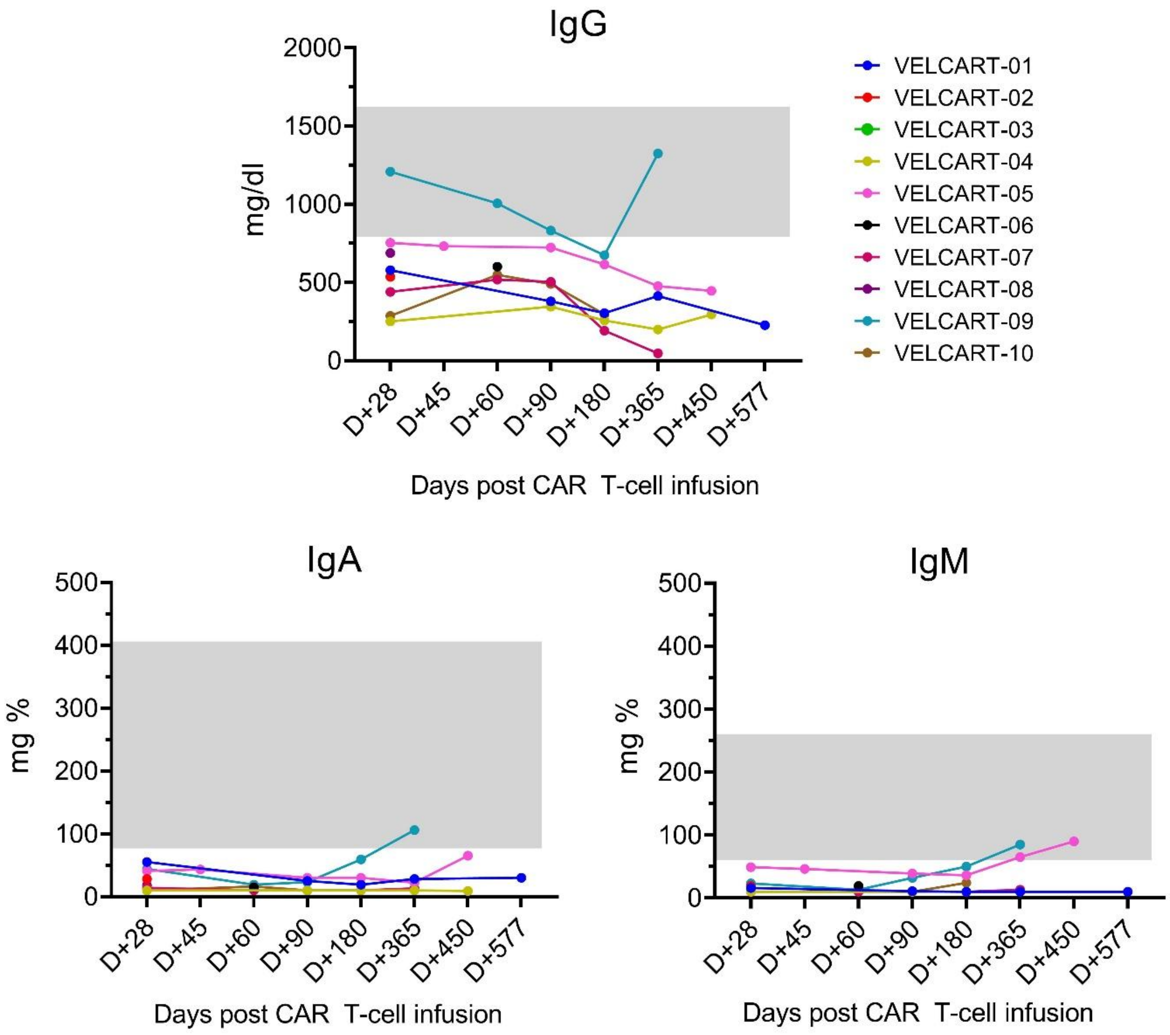

Figure S6. Serum immunoglobulin levels of patients post CAR T-cell infusion. Serum Immunoglobulin levels of IgG, IgA, and IgM in patients post CAR T-cell infusion. The shaded region indicates the normal reference ranges. The follow-up data was not available for VELCART 03 (died of progressive disease), VELCART 08 (withdrawn from study), and VELCART 02 and 06 (underwent HSCT).

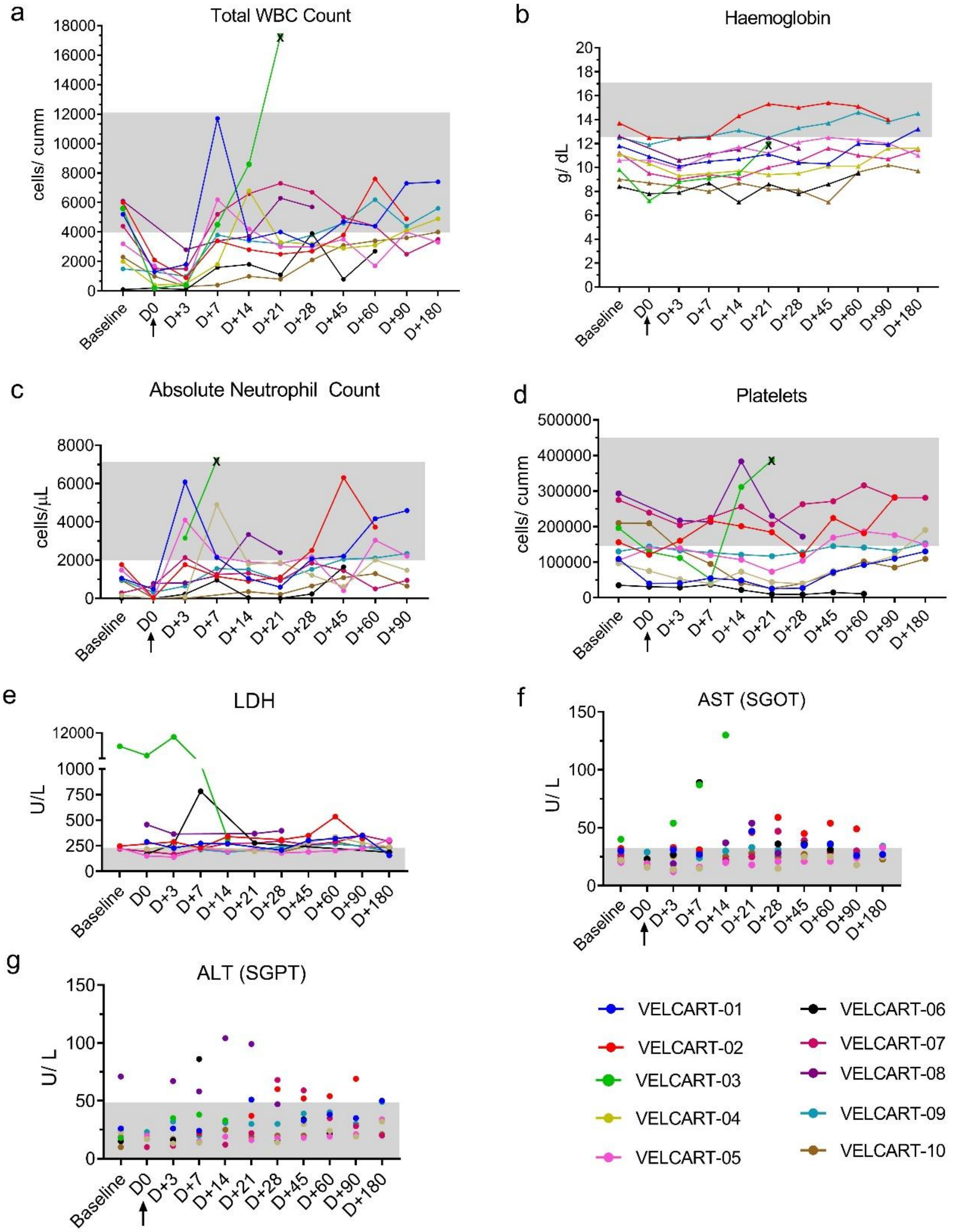

Figure S7. Patient demographics post CAR T-cell infusion. Peripheral blood assessments of cell count. LDH - Lactate Dehydrogenase, AST - aspartate aminotransferase, ALT - alanine aminotransferase. The shaded region indicates the normal reference ranges.

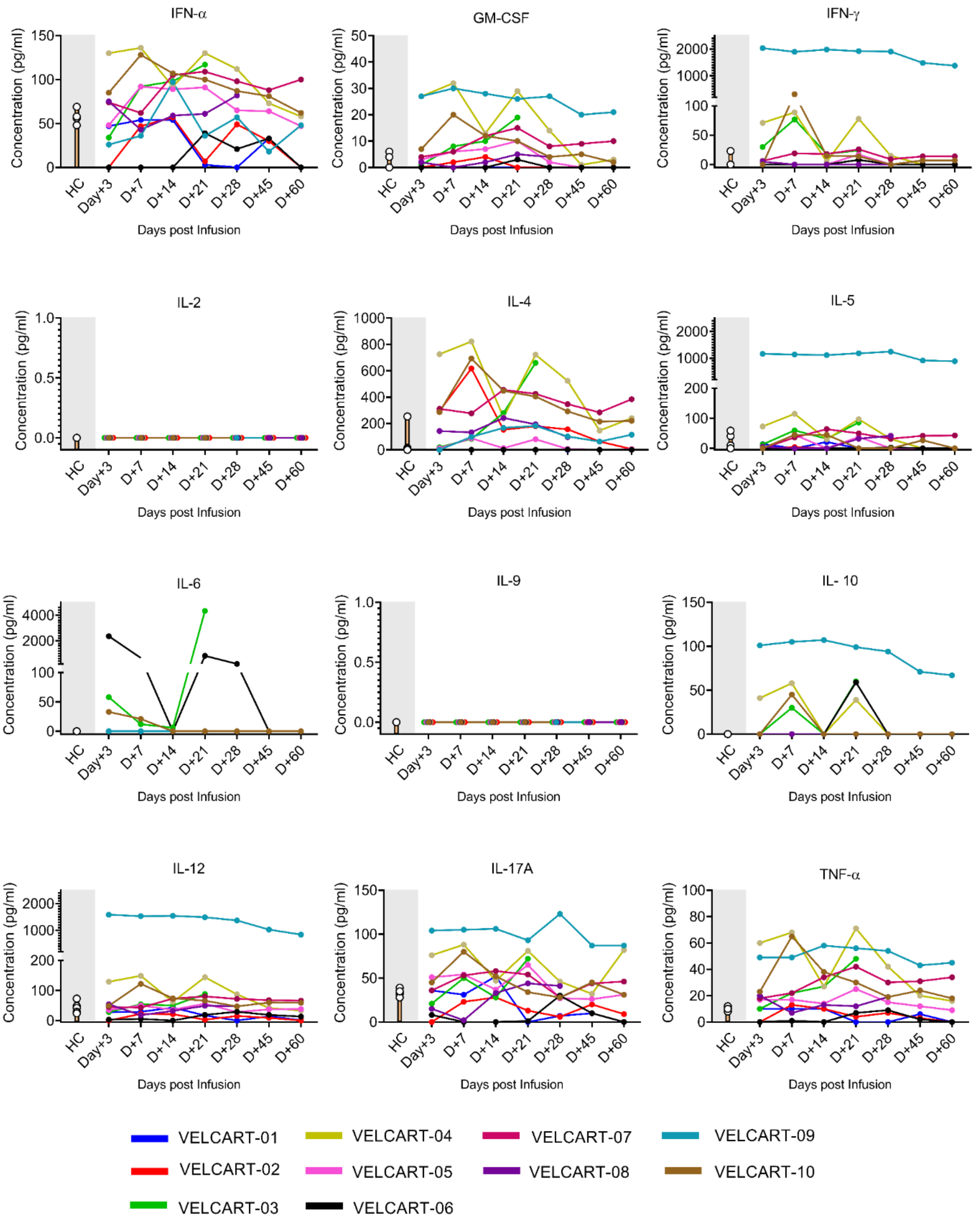

Figure S8. Plasma cytokines level assessments post CAR T-cell infusion. Cytokine levels of patients undergoing CAR T-cell therapy post-infusion in peripheral blood plasma. HC- Healthy controls (n=5).

Table S1. Patient's disease characteristics with treatment history

Patient	VELCART 01	VELCART 02	VELCART 03	VELCART 04	VELCART 05	VELCART 06	VELCART 07	VELCART 08	VELCART 09	VELCART 10
**Diagnosis**	Refractory B-ALL	Relapsed B-ALL	Relapsed refractory DLBCL	Relapsed refractory DLBCL	Refractory B-ALL	Relapsed refractory B-ALL	Relapsed B-ALL	Relapsed Refractory DLBCL	Refractory B-ALL	Primary refractory DLBCL
**Age & sex**	59 Male	20 Male	43 Male	59 Male	42 Male	29 Male	6 Male	48 Male	53 Male	57 Male
**No. of prior therapies & History**	4 LINES (Phase 1 Induction of GMALL Protocol - induction failure (bulk disease post-induction), three cycles of Mini HYPERCVAD + INO)	2 LINES: BFM 90 protocol - early medullary and CNS relapse - Mini Hyper CVAD+INO + TIT then one dose INO	2 LINES: 6 Cycles R-CHOP relapsed after 32 months - 5 Cycles RICE	6 LINES: RCHOP - relapsed after 29 months - RGCD, R-MINI BEAM, then Auto Tx done in PR f/b IFRT - then relapsed after 15 months - R-lenalidomide protocol	3 LINES: BFM 95 Adult ALL protocol phase 1 - induction failure (bulk disease post-induction), Phase 2 Induction, consolidation	3 LINES (BFM protocol - late medullary relapse - phase 1 induction - BM remission, testicular relapse - orchidectomy - MRD relapse - 2 cycles of INO)	3 LINES: (BFM protocol - early medullary relapse - BFM relapse protocol R1-R2-R1-VCR/ Bortezomib)	4 LINES: (R-CHOP - relapsed after 13 months - R-GDP, RICE, Auto Tx - relapsed after 8 months)	3 Lines: Adult ALL protocol: Received Phase-1, Received Phase-2, Inotuzumab x 3 doses	4 Lines: R-CHOP x 6 cycles, R-DHAP: 2 cycles, R-lenalidomide-Ibrutinib x 4 cycles, R-lenalidomide-ibrutinib-Ventoclax x 1 cycles, Polatuzumab, Bendamustine, Rituximab x 1 cycle
**Disease status at enrolment**	Residual disease	Residual disease	Progressive disease	Progressive disease	Residual disease	Bulk residual disease	Residual disease	Relapsed disease	Residual disease	Progressive disease

**MRD/ PET CT**	MRD: 0.17 %	MRD: 0.8 %	PET: Metabolically active findings: Small bowel, Jejunum, Ileum, Mesenteric nodes, left external Iliac nodes	PET: Metabolically active findings: Small bowel, Jejunum, Ileum, Mesenteric nodes, left external Iliac nodes, Left Inguinal nodes	MRD: 2.27 %	MRD: 28 %	MRD: 0.5 %	PET: Metabolic active findings: Sub-centric level 2 nodes, Left Para-aortic mass, Peri-portal node	MRD: 0.06 %	PET: Necrotic para-aortic nodes, Inguinal nodes, Abdominal wall, a superior component of epigastric region, antrum of the stomach, Lesion in Upper abdominal wall
**No prior lines of therapy (weeks)**	4	2	2	6	3	6	3	4	3	4
**Time from the last therapy (weeks)**	7	9	8	6	6	3	7	42	5	8

Table S2. CAR T-cell product release criteria assays

Details/ Assay	Method	VELCAR T 01	VELCAR T 02	VELCAR T 03	VELCAR T 04	VELCAR T 05	VELCAR T 06	VELCART 07	VELCART 08	VELCART 09	VELCART 10
**Diagnosis**	NA	Refractory B-ALL	Relapsed B-ALL	Relapsed refractory DLBCL	Relapsed refractory DLBCL	Refractory B-ALL	Relapsed refractory B-ALL	Relapsed B-ALL	Refractory DLBCL	Refractory B-ALL	Primary refractory DLBCL
**Age & sex**	NA	59 Male	20 Male	43 Male	59 Male	42 Male	29 Male	6 Male	48 Male	53 Male	57 Male
**No of days culture**	NA	9	9	9	9	9	9	9	9	9	9
**Viability**	Flow cytometry 7AAD	99 %	97 %	88 %	97 %	97 %	95 %	80 %	98 %	99 %	99 %
**Transduction efficiency**	Flow cytometry – CAR %	40 %	37 %	45 %	18 %	36 %	47 %	55 %	55 %	16 %	35 %
**Phenotype harvest**	Flow cytometry CD3 + cells	99 %	99 %	99 %	98 %	99 %	99 %	99 %	99 %	99 %	99 %
**Sterility**	Microbial culture	No growth	No growth	No growth	No growth	No growth	No growth	No growth	No growth	No growth	No growth
**Gram stain**	Grams's methods	Negative	Negative	Negative	Negative	Negative	Negative	Negative	Negative	Negative	Negative
**Endotoxin**	Endosafe PTS	<0.05 EU/ml	<0.05 EU/ml	<0.05 EU/ml	<0.05 EU/ml	<0.05 EU/ml	<0.05 EU/ml	<0.05 EU/ml	<0.05 EU/ml	<0.05 EU/ml	<0.05 EU/ml
**Mycoplasma**	PCR	Negative	Negative	Negative	Negative	Negative	Negative	Negative	Negative	Negative	Negative
**Vector copy number**	QPCR	2.0 copies/ cell	2.6 copies/ cell	1.8 copies/ cell	1.8 copies/ cell	1.4 copies/ cell	2.1 copies/ cell	2.0 copies/ cell	2.0 copies/ cell	2.0 copies/ cell	2.0 copies/ cell
**VSVG**	QPCR	UND	UND	UND	UND	UND	UND	UND	UND	UND	UND

Table S3. Sample process information

Sample details	Cell product volume (ml)	Concentration of WBC ($X10^6$/ml)	Total WBCs ($X10^9$)	Total labeled cells ($X10^9$)	Frequency of labeled cells (%)	Seeding count ($X10^6$)
VELCART 01	105	71.5	7.5	3.1	42.4	100
VELCART 02	100	100	10	7.2	72	
VELCART 03	100	45	4.5	1.4	33	
VELCART 04	180	31	5.5	3.5	62.4	
VELCART 05	190	111	21	13	61.9	
VELCART 06	200	19	3.8	2.9	76.2	
VELCART 07	140	33	4.6	1.7	37.2	
VELCART 08	180	130	23.4	8.9	38.4	
VELCART 09	150	72.5	10.8	3.7	34.5	
VELCART 10	189	47	8.8	3.9	44.5	

Table S4. Purity of apheresis sample and recovery post-enrichment of CD4+ and CD8+ cells

Sample details	% Among viable CD45+ cells			
	CD 4+ cells (%)		CD 8+ cells (%)	
	Pre-column purity	Post-column recovery	Pre-column purity	Post-column recovery
VELCART 01	14.6	34.8	14.7	34.3
VELCART 02	30.1	41.6	35.8	51
VELCART 03	11.3	33	17.4	44.5
VELCART 04	15.3	25.8	34	58.5
VELCART 05	40.3	64.4	13.7	27
VELCART 06	23.2	34.3	29.2	35
VELCART 07	22	70.5	7.2	21.5
VELCART 08	14.3	35.7	19.3	50.8
VELCART 09	9.1	27.6	17.2	60
VELCART 10	10	19.6	26	59.1

Table S5. Toxicities post CAR T-cell infusion comparing the dose levels

Sample details	Dose	CRS	ICANS	ICAHT	Hepatotoxicity	Nephro toxicity	Cardio toxicity
VELCART 01	0.5 X 10^6/ Kg	Grade 1	Nil	Grade 1	Nil	Nil	Nil
VELCART 02	0.5 X 10^6/ Kg	Grade 2	Nil	Grade 1	Grade 1	Nil	Nil
VELCART 03	0.5 X 10^6/ Kg	Grade 1	Nil	Grade 1	Grade 2	Nil	Nil
VELCART 04	1 X 10^6/ Kg	Grade 1	Nil	Grade 2	Nil	Nil	Nil
VELCART 05	1 X 10^6/ Kg	Grade 1	Nil	Grade 1	Nil	Nil	Nil
VELCART 06	1 X 10^6/ Kg	Grade 3	Nil	Grade 3	Nil	Nil	Nil
VELCART 07	2 X 10^6/ Kg	Grade 1	Nil	Grade 1	Grade 1	Nil	Nil
VELCART 08	2 X 10^6/ Kg	Grade 1	Nil	Grade 1	Nil	Nil	Nil
VELCART 09	2 X 10^6/ Kg	Grade 1	Nil	Grade 1	Nil	Nil	Nil
VELCART 10	2 X 10^6/ Kg	Grade 1	Nil	Grade 2	Nil	Nil	Nil

Supplemental Methods

Clustering analysis

Different T-cell subsets were identified and clustered by comparing the B-ALL and DLBCL samples based on their marker expression profiles. Naïve (CD27+ CD45RA+), Central memory (CD27+CD45RA-), Effector memory (CD27-CD45RA-), Terminally differentiated (CD27-CD45RA+). T-cells were identified with CD3 after sequentially excluding doublets, dead cells, CD45-negative events, B-cells, and monocytes. Unsupervised clustering via FLOWSOM, after dimensionality reduction through UMAP, was performed on 400,000 T-cells (200,000 events from leukemia and lymphoma samples) using FlowJo™ software (BD Biosciences).
