# Supplementary material for: Safety, efficacy and total cost of point-of-care manufactured anti-CD19 CAR-T cell therapy in India: VELCART trial
Source: Mol Ther Oncol. 2025 Mar 25;33(2):200977. doi: 10.1016/j.omton.2025.200977 (PMC12005290; doi:10.1016/j.omton.2025.200977)
Supplement: Document S2. Article plus supplemental information [file mmc2.pdf]

Molecular Therapy
Oncology
Original Article

Safety, efficacy and total cost of point-of-care manufactured anti-CD19 CAR-T cell therapy in India: VELCART trial

Hamenth Kumar Palani,[1] Arun Kumar Arunachalam,[1] Uday Kulkarni,[1] Mohammed Yasar,[1] Arvind Venkatraman,[1] Swathy Palanikumar,[1] Reeshma Nair Radhakrishnan,[1] Majeela Solomon,[1] Abirami Rajasekaran,[1] Aniket Bankar,[2] Phaneendra Venkateswara Rao Datari,[1] Sushil Selvarajan,[1] Anu Korula,[1] Pradyot Dash,[3] Dina Schneider,[3] Louisa Wirthlin,[3] Aby Abraham,[1] Biju George,[1] and Vikram Mathews[1]

[1]Department of Haematology, Christian Medical College, Ranipet Campus, Vellore 632517, Tamil Nadu, India; [2]Princess Margaret Cancer Center, University Avenue, Toronto, ON M5G2C1, Canada; [3]Lentigen Technology Inc., A Miltenyi Biotec Company, Gaithersburg, MD 20878, USA

Decentralized or point-of-care (POC) manufacture of CAR-T cells is a potential strategy to improve accessibility and reduce cost and logistic challenges. A total of 10 relapsed/refractory patients (B cell acute lymphoblastic leukemia [B-ALL] $N = 6$, diffuse large B cell lymphoma [DLBCL] $N = 4$) were enrolled in this POC phase 1 study. Chimeric antigen receptor (CAR)-T cells were manufactured using the fully automated CliniMACS Prodigy system. The CAR-T cell products had a median 15-fold expansion with a median transduction rate of 38%. The immunophenotypic characterization indicates a significant increase in central memory and effector T cells. All the patients were infused with fresh CAR-T cells. Complete remission rates were 100% for B-ALL and 50% for DLBCL. At a median follow-up of 15 months, 8 of 10 patients remain without disease progression. Adverse events reported were cytokine release syndrome grade 2 or higher in 2 of 10 patients. None of the patients developed immune effector cell-associated neurotoxicity syndrome. Late hematological toxicity of grade 2 or higher was noted only in one patient. Evaluation of health care resource utilization demonstrates that the median cost was US$12,724, while the manufacturing cost was US$35,107. Our data highlight the safety, efficacy, low cost, and potential to enhance the accessibility of CAR-T cell therapy in low- and middle-income countries through a fully automated and closed manufacturing platform.

INTRODUCTION

Chimeric antigen receptor (CAR) T cell therapy targeting CD19 is an effective therapeutic modality against chemo-refractory B cell leukemia and lymphoma.[1,2] CARs are engineered fusion proteins consisting of antigen recognition and T cell activation domains that redirect T cells to recognize and eliminate cells that specifically express the target antigen(s). With its remarkable clinical success and U.S. Food and Drug Administration approval of several CAR-T cell products,[3] this therapy has rapidly gained the standard of care status in relapsed/refractory (r/r) B cell leukemia and lymphoma. However, access to CAR-T cell therapy is limited by cost and turnaround time, even in developed countries.[4,5] The current centralized model with industry-driven CAR-T cell manufacturing is not viable to the requirements and realities of developing countries like India.[6] Moreover, the challenges faced by a clinical team treating a cohort of patients requiring urgent therapeutic decisions and experiencing rapid changes in clinical status makes it difficult to work with a centralized system with an uncertain manufacturing schedule, even in developed countries.[7] With the existing centralized manufacturing and logistics model, it is estimated that only 25% of the patients registered for CAR-T cell infusion are likely to receive it. The median wait time for a patient on such lists is 6 months.[7] Considering the cost of the product and therapy, it is unlikely that the current centralized and industry-driven manufacturing model will overcome these challenges.

Decentralized or point-of-care (POC) manufacturing using fully automated processing systems provides an alternate option to this centralized model, which has proven feasibility and efficacy.[8,9] In addition to lowering production costs, it reduces the expenses and logistical challenges related to cryopreservation, stringent shipping, and cold chain requirements.[10] Additionally, data suggests that a fresh product is likely to be more viable and exhibits more significant and faster expansion *in vivo*, potentially translating to increased efficacy.[11]

We have previously reported on decentralized CAR-T cell manufacturing using the CliniMACS Prodigy system (Miltenyi Biotec) through our pre-clinical validation runs.[12] We also evaluated the cost associated with this manufacturing process. This study builds on

Received 6 January 2025; accepted 21 March 2025;
https://doi.org/10.1016/j.omton.2025.200977.

Correspondence: Vikram Mathews MD, DM, FASc Professor, Department of Haematology, Christian Medical College, Ranipet campus, Vellore 632 517, Tamil Nadu, India.
E-mail: vikram@cmcvellore.ac.in

© 2025 The Author(s).
Published by Elsevier Inc. on behalf of The American Society of Gene and Cell Therapy.
This is an open access article under the CC BY license (http://creativecommons.org/licenses/by/4.0/).

Table 1. Baseline demographics and disease characteristics of patients treated with anti-CD19 CAR-T cell therapy

Clinical parameters	B-ALL (*N* = 6)	DLBCL (*N* = 4)
Age (years)	35 (2–59)	52 (43–59)
Sex	male (100%)	male (100%)
Hemoglobin (gm %)	11.7 (10.0–13.8)	10.4 (8.8–12.8)
White blood cells total (/μL)	4,700 (2200–10300)	3950 (2000–8100)
Absolute lymphocyte count (/μL)	915 (840–2142)	1030 (840–2430)
Platelet count (/μL)	1 83 000 (52 000–2 62 000)	2 41 500 (1 14 000–2 81 000)
Liver function test		
Total bilirubin (mg/dL)	0.32 (0.24–1)	0.59 (0.16–0.74)
Serum glutamic oxaloacetic transaminase (U/L)	27 (20–36)	29.5 (14–47)
Serum glutamic-pyruvic transaminase (U/L)	23 (20–47)	23 (12–72)
Serum creatinine (mg %)	0.49 (0.3–0.69)	0.79 (0.36–1.07)
Serum LDH (U/L)	190 (151–288)	420 (219–11 120)
Baseline Karnofsky score (%)	90	90
Patients with additional comorbidities		
Diabetes mellitus	1	0
Cardiovascular disease	2	1
Others[a]	0	0
No. of prior lines of therapy	3 (2–4)	3 (2–6)
Bone marrow blast (%)	0.65 (0.06–28)	NA
CNS or other extramedullary involvement before lymphodepletion	Nil	Nil
Time from last therapy (weeks)	6.5 (3–9)	8 (6–48)
Total CAR-T cell dose ($\times 10^6$)	52 (29.5–86)	93 (20.5–134)

LDH, lactate dehydrogenase; SGOT, serum glutamic-oxaloacetic transaminase; SGPT, serum glutamate pyruvate transaminase.
[a]Other comorbidities include cerebrovascular disease, obesity, rheumatologic disease, renal dysfunction, pulmonary dysfunction, psychiatric conditions, and prior malignancies.

that experience and presents the results of our phase 1 clinical trial using on-site-manufactured CAR-T cells to treat r/r B cell leukemia and lymphoma patients.

While the cost of CAR-T cell therapy is often discussed regarding the manufacturing and delivery of CAR-T cells, it is well recognized that the cost of supportive care before and after CAR-T cell infusion can sometimes be as high as the cost of the product. This includes the cost of apheresis, bridging therapy, and management of complications such as cytokine release syndrome (CRS)/immune effector cell-associated neurotoxicity syndrome (ICANS) and continued follow-up and monitoring of patients. There are limited data on the total cost of CAR-T cell therapy, and it is not frequently reported in the context of clinical trials. It is estimated that the post-infusion costs can be around US$150,000, and the management of patients with severe CRS could reach one-half of a million dollars.[13,14]

Our study aims to evaluate the safety, efficacy, and total cost of these POC-manufactured CAR-T cells. This is the first clinical study in India to assess the feasibility of a POC-manufactured CAR-T cell therapy process, including health care resource utilization (HRU) analysis.

RESULTS

Manufacturing anti-CD19 CAR-T cells using Prodigy

A total of 10 patients were enrolled in this dose escalation phase 1 clinical trial comprising of 6 r/r B cell acute lymphoblastic leukemia (B-ALL) (VELCART 01, 02, 05, 06, 07, and 09) and 4 diffuse large B cell lymphoma (DLBCL) (VELCART 03, 04, 08, and 10) with a median age of 45 years (range, 6–59 years). Patients had a median of three (range, 2–6) prior lines of therapy. In the B-ALL cohort, five of the six had refractory residual disease with detectable measurable residual disease (MRD) by flow cytometry (0.06%–2.27%), and one had r/r and bulk disease with 28% blasts in the marrow. In the lymphoma cohort, one of the four had primary refractory disease, while the rest had relapsed and refractory to progressive disease. Detailed patient demographics with treatment history are summarized in Tables 1 and S1.

Clinical grade CAR-T cells were produced in house with a vein-to-vein time of 9 days using the CliniMACS Prodigy system (Figure 1A). The study was designed to infuse fresh CAR-T cells at the end of manufacture. Apheresis was started on day −9, and the first quality check (QC) on day −6, assessing transduction efficiency and cell counts, provides a go or no-go signal for admitting the patient for

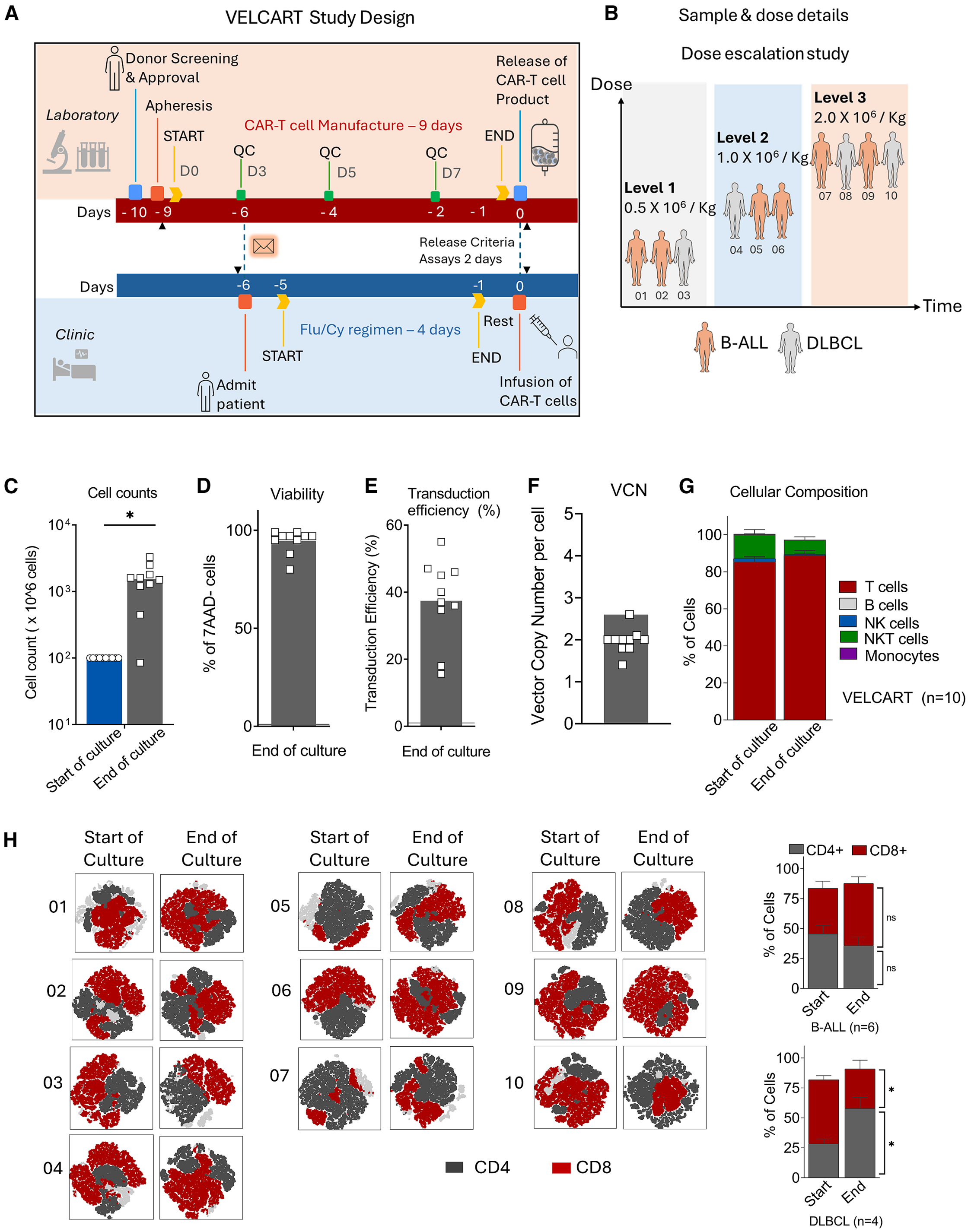

Figure 1. Workflow and characterization of anti-CD19 CAR-T cells manufactured using the Prodigy system

(A) The workflow diagram illustrates the study design with the timeline of the CAR-T cell manufacturing process and the preparation of patients for a fresh infusion of CAR-T cells. (B) $CD19^+$ B-ALL (n = 6) and DLBCL (n = 4) were included in the study. The patients were infused with an increasing dose at different levels, starting from 0.5×10^6/kg (N = 3), 1.0×10^6/kg (N = 3), and 2.0×10^6/kg (N = 4). (C) Fold expansion of CAR-T cells comparing the start and end of culture (n = 10). (D) The viability of the final CAR-T cell

(legend continued on next page)

fludarabine and cyclophosphamide conditioning before the infusion. All the release criteria assays were done on the day −2 QC sample. The release criteria values and descriptions details are provided in Table S2. On day 0, the final product was released after flow cytometric assessments and if the cultures remained negative.

CAR-T cell product manufacture was successful for all patients. All the patients were infused with fresh CAR-T cells. The study was a 3 + 3 dose escalation strategy started with the cell dose 0.5 × 10^6/kg (N = 3), 1 × 10^6/kg (N = 3), and 2 × 10^6/kg (N = 4) (Figure 1B). The median culture expansion was 15-fold (1-33) with a viability of 97% ± 5% (Figures 1C and 1D). The median transduction efficiency was 38% (range, 16%–55%), with the mean vector copy number (VCN) being 1.97 ± 0.3 copies per CAR-T cell (Figures 1E and 1F). The replication-competent lentivirus was not detected in any of the products. The purity of CAR-T cell products was evaluated by flow cytometry, showing $CD3^+$ T cells with a median of 98% (range, 98%–100%). No $CD19^+$ B cells were detected in the enriched T cells and final infused product (Figure 1G). In contrast with the B-ALL CD4/CD8 ratio median of 0.6 (range, 0.3–1.9), the DLBCL products had an increased ratio of 2.3 (range, 0.5–3.2) (Figure 1H).

Phenotyping of the starting T cells and final CAR-T cell product

The sample process information and the details of post-enrichment recovery of $CD4^+$/$CD8^+$ T cells were summarized in Tables S3 and S4. Eight of the 10 patients had more CAR-T cells than the required cell dose for infusion, and the additional cells were cryopreserved according to standard protocols. To assess the efficacy of cryopreservation, the samples were thawed and analyzed after 6 months. The products had a median viability of 91% (range, 85%–93%) (Figure S1). Immunophenotype of the T cells was done in the starting apheresis sample to identify the T cell subsets and differentiation based on expression of CD45RA and CD27. Both manual gating and unsupervised clustering revealed differences in B-ALL and DLBCL samples with a more naive phenotype in B-ALL patients and an increased effector memory T cells in DLBCL patients (Figure S2).

Consistent with clustering analysis, our previous pre-clinical validations[12] and even other studies using the same lentiviral vector,[15] T cell differentiation analysis in the final CAR-T cell products demonstrated a significant increase in central memory T cells ($CD45RO^+$ $CD62L^+$) in both leukemia and lymphoma across the subsets (Figure 2A). However, the effector memory ($CD45RO^+$ $CD62L^-$) cells were decreased in the CD4, with no difference in the $CD8^+$ T cells. Additionally, the percentage of effector cells ($CD45RO^-$ $CD62L^-$) significantly reduced in the $CD8^+$ T cells compared with baseline. Both subsets showed reduced naive cells ($CD45RO^-$ $CD62L^+$) in the final CAR-T cell product (Figures 2B, S3, and S4).

Analysis of exhaustion markers in the released product revealed a significant increase in $LAG3^+$ cells in a DLBCL, especially in CAR^+CD8^+ subsets compared with B-ALL. This was most marked in VELCART 03, who was a heavily pretreated patient with a progressive disease and had lower cell expansion. However, other exhaustion markers like PD1 and TIM3 were prominent by their absence in the manufactured CAR-T cells (Figure 2C).

Anti-tumor activity of manufactured CAR-T cells

To study the effector functions of manufactured CAR-T cells, we assessed the cytokine levels in the supernatants exposed to target ($CD19^+$ cells NALM6) and non-target cells ($CD19^-$ cells K562). The CAR-T cells exposed to the target produced a significant increase in Th1 cytokine interferon (IFN)-γ, granulocyte-macrophage colony-stimulating factor (GM-CSF), interleukin (IL)-2, and tumor necrosis factor (TNF)-α along with a marginal increase in T helper type 2 cytokines like IL-4, IL-5, and IL-10 for the survival, anti-tumor response and other pro-inflammatory functions (Figure 3A).

The antitumor activity of CAR-T cells was evaluated using a flow cytometry-based *in vitro* cytotoxicity assay, in which the CAR-T cells were exposed to target or non-target cells in different ratios after the viability analysis. Significant cell lysis was observed in all effector-to-target ratios compared with non-target cells, indicating the antigen-specific cytotoxicity of anti-CD19 CAR-T cells (Figure 3B). Additionally, the activation of CAR-T cells was confirmed by the expression of CD25 and CD137 markers. However, CD154 remained negative (Figure 3C). These CAR-T cells were re-stimulated with target cells every 48 h for 6 days to evaluate their potency. The re-stimulated CAR-T cells showed increased proliferation with minimal exhaustion (Figure S5), demonstrating the antigen-specific effector function and proliferation (Figure 3D). There was no comparable difference between the anti-tumor activity of CAR-T cells derived from B-ALL and DLBCL patients.

Efficacy of CAR-T cells post infusion, toxicities and characteristics

All patients were infused with fresh CAR-T cells. Among 10 patients evaluated for response, all six B-ALL patients (100%) achieved complete remission (CR), and all were MRD negative by day 90. In contrast, among the patients with DLBCL, two of the four (50%) achieved CR; one patient had a partial response (PR), and another patient in the first cohort with the lowest dose did not respond and died early due to progressive disease on day 21 (Figure 4A). The assessment of disease as per the protocol defined timelines in B-ALL (Figure 4B) and DLBCL, where one of the patients is illustrated in Figure 4C. At a median follow-up of 15 months (range, 13–21 months), 8 of the 10 patients remain without disease progression. In the B-ALL cohort, 2/6 patients underwent hematopoietic

product was assessed using 7AAD staining by flow cytometry (n = 10). (E) Transduction efficiency of the final CAR-T cell product by CD19 CAR detection reagent. (F) qPCR analysis of CAR-T cell product's vector copy number (VCN) per cell before infusion. (G) The cellular composition of the CAR-T cell product is based on flow cytometric characterization compared with the starting culture. (H) t-distributed Stochastic Neighbor Embedding plots demonstrate CAR-T cell products' CD4 and CD8 ratios by comparing their initial culture. Data represents mean ± SEM, * $p < 0.05$, ns: not significant. An unpaired two-tailed Student's t test was used for statistical analysis.

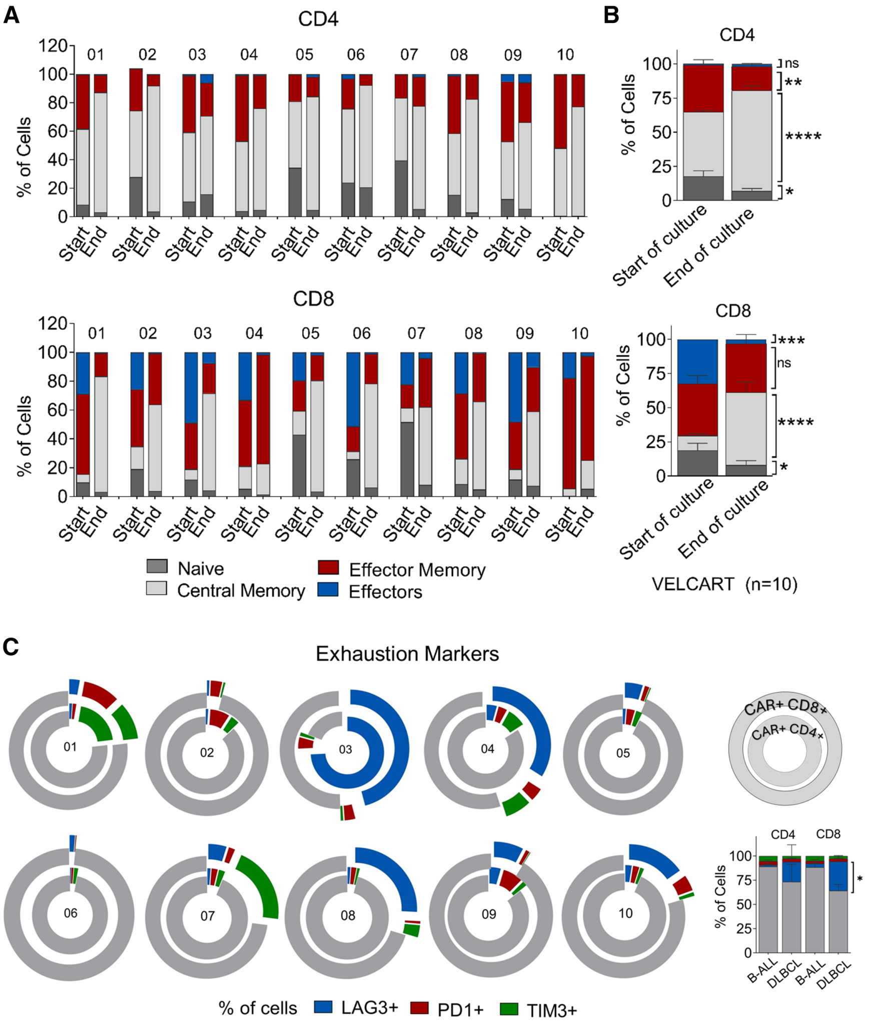

Figure 2. Immunophenotypic characterization of CAR-T cells

(A) Differentiation subsets of manufactured CAR-T cells with their percentage of cells in CD4 and CD8 subsets comparing the starting T cell culture and final CAR-T cell products based on the expression of markers CD45RO and CD62L. Naive ($CD45RO^-$ $CD62L^+$), central memory ($CD45RO^+$ $CD62L^+$), effector memory ($CD45RO^+$ $CD62L^-$), and effectors ($CD45RO^-$ $CD62L^-$). (B) Combined analysis of starting T cells and final product with a statistical analysis comparing the starting culture with their respective subset populations. (C) Analysis of exhaustion markers in the final infused CAR-T cell product. The double donut plot demonstrates the expression of exhaustion markers LAG3, PD1, and TIM3 as slices among the CAR^+ $CD4^+$ in the inner ring and CAR^+ $CD8^+$ in the outer ring. Each number represents a patient. Data represents mean ± SEM, * $p < 0.05$, ** $p < 0.01$, *** $p < 0.001$, **** $p < 0.0001$, ns: not significant. An unpaired two-tailed Student's t test was used for statistical analysis.

stem cell transplantation (HSCT) on day +76 and day +60. Both patients remained MRD negative post HSCT; one of them died on day +152 from graft-versus-host disease. The outcomes of the post-CAR-T cell infusion are summarized in Table 2.

Toxicities or adverse events of special interest reported were CRS grade 1, 80% (8/10); with CRS grade 2 (10%) and CRS grade 3 (10%) reported in one patient each. None of the patients developed ICANS of any grade (Figure 4D). Early hematological toxicity (immune effector cell-associated hematotoxicity [ICAHT]) was common, and 3 of the 10 patients (30%) had grade 2 or higher neutropenia and late hematological toxicity of grade 2 or higher was observed only in 1 patient (Figure 4E). None of the patients developed any dose-limiting toxicity per dose level, as summarized in Table S5. CAR-T cell persistence by flow cytometry demonstrated a maximum increase in CAR^+ T cells in peripheral blood mononuclear cells on day 18.[7–27] Cell dose and malignancy type did not seem to influence CAR-T cell proliferation or persistence (Figure 4F). The phenotypic composition of persistent CAR^+ cells was predominantly $CD8^+$ with increasing effector memory and effector T cells over time, demonstrating the antitumor activity followed by a gradual increase in naive and central memory cells (Figure 4G).

Immune reconstitution profile in patients post CAR-T cell infusion

The lymphocyte subset analysis confirmed that the lymphocytes recovered within 14–28 days post infusion, where the natural killer (NK) cell was first to achieve normal levels within 30 days (range, 14–28 days), with a consistent increase over time (Figure 5A). The B cell aplasia persisted in all patients until day +90, with two ALL patients (VELCART 05 and 09) showing circulating B cells at later points (day +180 and day +365), but remaining in remission until the last follow-up (15 and 12 months, respectively). Similarly, hypogammaglobulinemia was noted in 9 of 10 patients beyond 1 year, except VELCART 09, who showed increased IgG levels at day +365 (Figure S6). Consistent with CAR^+ T cells kinetics, the total T cell subsets showed a similar recovery pattern with immediate recovery of $CD8^+$ T cells compared with $CD4^+$ T cells. The memory subsets demonstrate the rapid expansion of effector memory T cells followed by a transient increase in central memory T cells (Figures 5B and 5C); the data are similar to previous studies using different CAR-T cell products.[16,17]

The patient's peripheral blood cell characterization demonstrated the transient rise and subsequent normalization of liver enzymes, lactate dehydrogenase, cytokines, and the gradual normalization of blood counts post-CAR-T cell infusion, as illustrated in Figures S7 and S8.

HRU and total cost of CAR-T cell therapy

Despite its promising treatment outcomes, access to CAR-T cell therapy is limited by its cost, especially in low- and middle-income

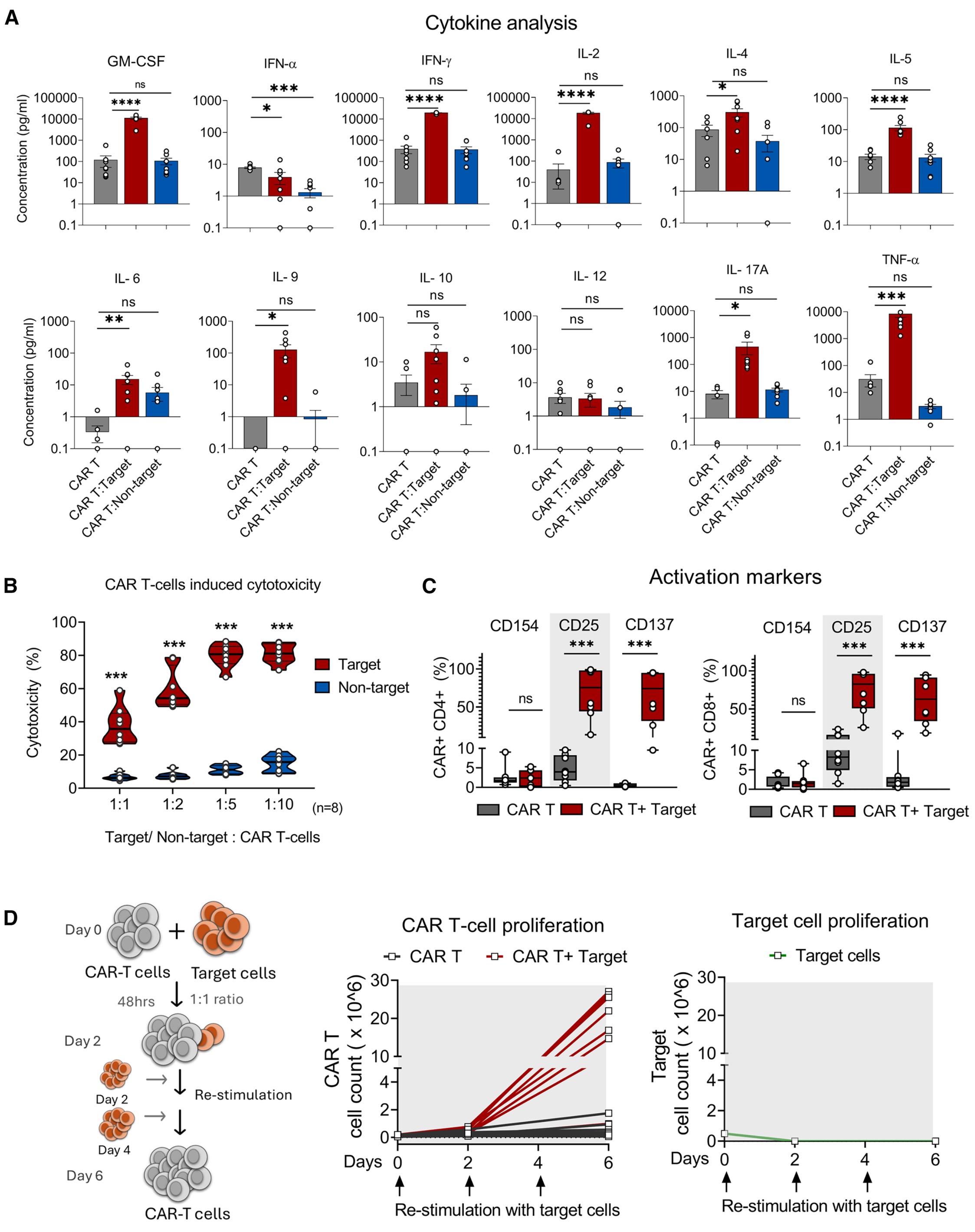

Figure 3. Functional analysis of CAR-T cells

(A) Cytokine analysis of CAR-T cells exposed to target cells ($CD19^+$ cells – NALM6) and non-target cells ($CD19^-$ cells – K562) from the supernatants evaluated after overnight incubation. (B) Cytotoxic activity of CAR-T cells exposed to target or non-target cells at different ratios starting from 1:1 to 1:10. The viability was assessed by 7AAD staining after overnight incubation by flow cytometry. (C) The activation of CAR-T cells was evaluated after 24 h of incubation with target cells by analyzing CD154, CD25, and CD137 among CAR^+ $CD4^+$ cells and CAR^+ $CD8^+$ cells. (D) Expansion of CAR-T cells after antigen-specific stimulation, where CAR-T cells were co-cultured with an equal number of target cells with intermittent stimulation for 6 days. The proliferation of CAR T and target cells was assessed by flow cytometry using CAR detection reagent (CAR-T cells) and CD19 expression (target cells NALM6) (n = 8). Each dot represents each sample. Data represents mean ± SEM, * $p < 0.05$, ** $p < 0.01$, *** $p < 0.001$, **** $p < 0.0001$, ns- not significant. An unpaired two-tailed student t-test was used for statistical analysis.

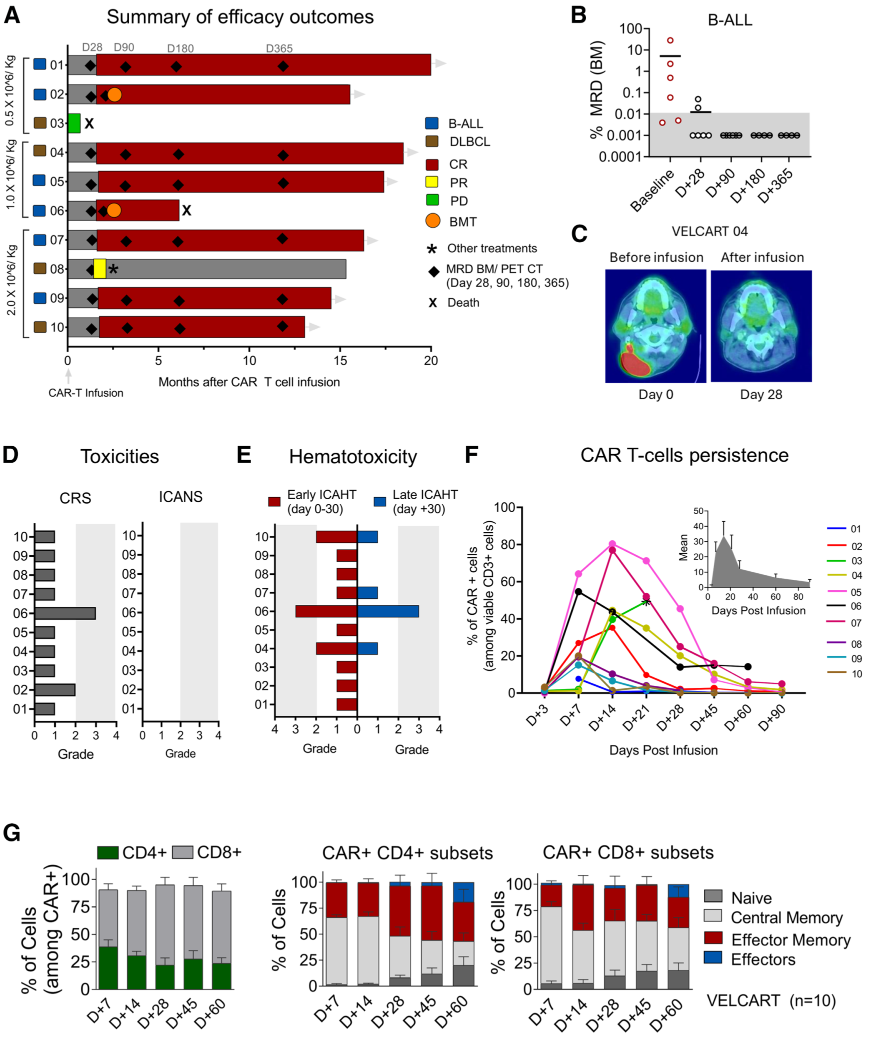

Figure 4. Efficacy of CAR-T cells post-infusion, toxicities, and characteristics

(A) Swimmer plot demonstrates the efficacy of CAR-T cells post-infusion in patients. The horizontal bar corresponds to each patient (B-ALL *N* = 6, DLBCL *N* = 4). CR, red; PD, progressive disease, green; PR, yellow . Each diamond indicates the assessment of leukemia/tumor burden by MRD via flow cytometry in leukemia and PET CT scan for lymphoma. The patients who underwent bone marrow transplantation (BMT) were denoted as orange circles. (B) Bone marrow MRD assessment by flow cytometry on B-ALL patients post CAR-T cell infusion. The gray area indicates undetectable ALL cells. (C) Whole-body fludeoxyglucose-18 (FDG) PET-CT imaging performed 28 days after CAR-T cell infusion in patient VELCART 04 with DLBCL showed a marked reduction in FDG avidity in the neck compared with before infusion. (D) The bar graph demonstrates the toxicities of CAR-T cells CRS and ICANS. (E) ICAHT grading based on all patients' depth and duration of neutropenia. (F) Detection of CAR-T cells in the patient's peripheral blood post-infusion by flow cytometric assessment demonstrating the percentage of CAR-positive cells among gated $CD3^+$ T cells at appropriate days post CAR-T cell infusion. The mean increase of CAR^+ cells on days 15–20 post infusion. (G) Immunophenotypic characterization of circulating CAR-T cells demonstrating the ratio of CD4 and CD8 among $CD3^+CAR^+$ cells following the subsets of T cells naive ($CD45RO^-CD62L^+$), central memory ($CD45RO^+$ $CD62L^+$), effector memory ($CD45RO^+$ $CD62L^-$) and effectors ($CD45RO^-CD62L^-$).

countries like India. Hence, we evaluated the total cost of patients undergoing CAR-T cell therapy, incorporating HRU and manufacturing costs as described previously, along with the cost of administration and care of patients post-CAR-T cell infusion. The retrospective analysis of the activity-based micro-costing approach was used for computation from the hospital perspective. The median hospitalization of the patients was 20 days (range, 15–37 days) for CAR-T cell infusion. The HRU was assessed 7 days before infusion and 60 days (median) post infusion for inpatient and outpatient visits. The costing data were calculated from the hospital accounting system and may differ from that of other hospitals.

The Sankey diagram illustrates the cost of health care resources during the entire treatment of CAR-T cell therapy, which comprises inpatient and post-CAR T outpatient care. The mean expenditure of 10 patients was calculated to analyze the cost drivers. Apheresis, hospital care, laboratory investigations, medications, consultancy, and transfusions were the major cost drivers to the total cost of $US12,724. Post-CAR T outpatient cost was factored into the in-patient cost together to determine the total cost of therapy (Figure 6A). We have previously demonstrated the manufacturing cost per product based on a micro-costing analysis of US$35,107, excluding the cost of the lentiviral vector[12] (Figure 6B). Together with our CAR-T cell production cost, this study's post-manufacturing cost analysis for HRU/clinical management per patient for leukemia and lymphoma, including intensive care unit admission and management of adverse events and follow-up, demonstrated that the mean cost was US$47,831 (excluding the cost of the lentiviral vector) (Figure 6C).

DISCUSSION

This phase 1 study demonstrates the successful establishment and safe delivery of a POC-manufactured CAR-T cell product. This process offers various advantages over centralized manufacturing, such as eliminating the need for bridging therapy, reducing vein-to-vein time, and allowing for the infusion of fresh products. The CAR-T cells manufactured using the CliniMACS Prodigy system showed a 15-fold expansion with a median transduction efficiency of 38% and viability of 97%. The CAR-T cell final product had varied

Table 2. Summary of patient outcomes post CAR-T cell infusion

Patient	VELCART 01	VELCART 02	VELCART 03	VELCART 04	VELCART 05	VELCART 06	VELCART 07	VELCART 08	VELCART 09	VELCART 10
Diagnosis	refractory B-ALL	relapsed B-ALL	r/r DLBCL	r/r DLBCL	refractory B-ALL	r/r B-ALL	Relapsed B-ALL	refractory DLBCL	refractory B-ALL	primary refractory DLBCL
Age	59	20	43	59	42	29	6	48	53	57
CAR-T cell dose	0.5×10^6/kg	0.5×10^6/kg	0.5×10^6/kg	1×10^6/kg	1×10^6/kg	1×10^6/kg	2×10^6/kg	2×10^6/kg	2×10^6/kg	2×10^6/kg
CRS	grade 1	grade 2	grade 1	grade 1	grade 1	grade 3	grade 1	grade 1	grade 1	grade 1
ICANS	nil	nil	nil	nil	nil	nil	nil	nil	nil	nil
MRD/PET CT (day 28)	negative	negative		CR	negative	0.02%	0.05%		negative	CR
MRD/PET CT (day 90)	negative	day 76: negative (proceeded to BMT)		CR	negative	day 60: MRD negative (proceeded to BMT)	negative		negative	CR
MRD/PET CT (day 180)	negative	NA	died on day +20 due to disease progression	CR	negative	post CAR T day +60, proceeded to BMT. died on day +152 to GVHD	negative	PR and subsequent progression on palliative care	negative	CR
MRD/PET CT (day 365)	negative	NA		CR	negative		negative		negative	CR
Days post infusion (17-12-24)	643	post CAR-T cell day +76, proceeded to HSCT day +464		552	522		489		433	391
Survival status	alive	alive	Died	alive	alive	Died	alive	alive	alive	alive

BMT, bone marrow transplantation; GVHD, graft-versus-host disease.

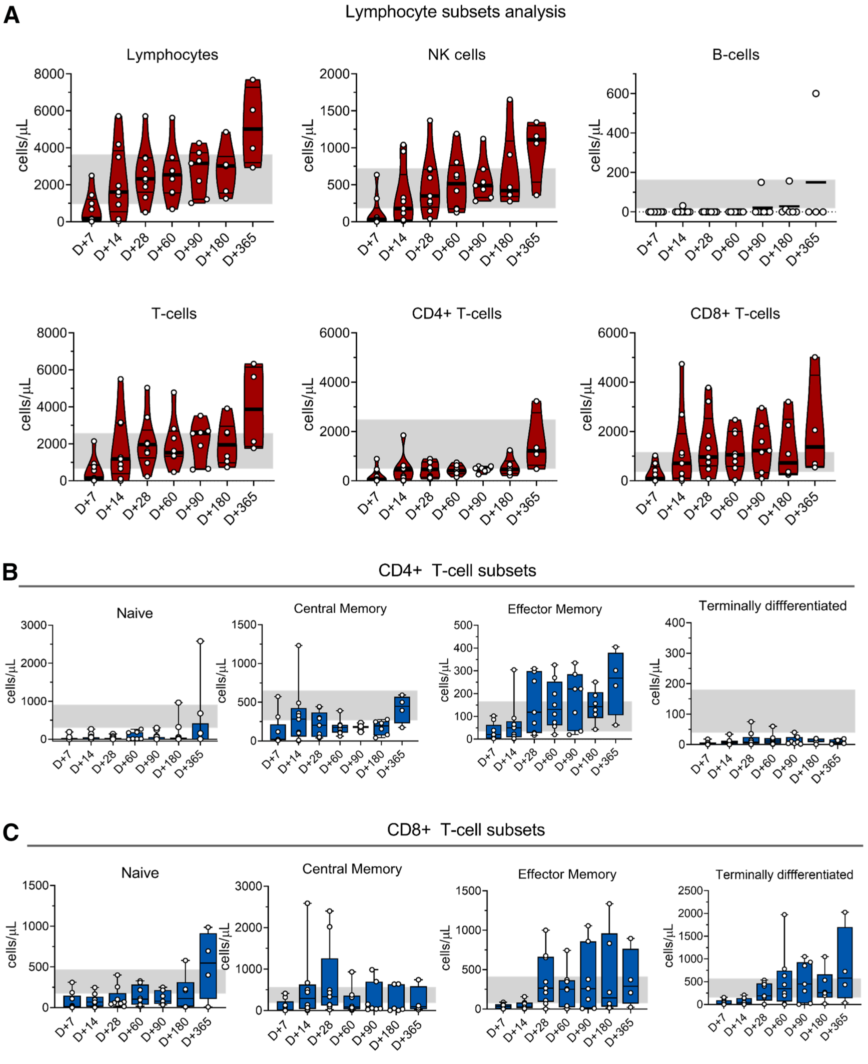

Figure 5. Immune reconstitution profile in patients post CAR-T cell infusion

(A) The graph demonstrates the recovery of lymphocyte subsets on follow-up days post CAR-T cell infusion in patients by flow cytometry. The date represents the absolute counts of cells per microliter. Lymphocytes (CD45 bright), B cells ($CD45^+$ $CD19^+$), NK cells ($CD45^+$ $CD3^-$[CD16 and CD56] $^+$), T cells ($CD45^+$ $CD3^+$), CD4 T cells ($CD3^+$ $CD4^+$ $CD8^-TCR\gamma\delta^-$), CD8 T cells ($CD3^+$ $CD4^-$ $CD8^+$ $TCR\gamma\delta^-$). (B) The $CD4^+$ T cell subset in patients Naive ($CD27^+CD45RA^+$), Central memory ($CD27^+$ $CD45RA^-$), effector memory ($CD27^-$ $CD45RA^-$), terminally differentiated ($CD27^-CD45RA^+$). (C) The subsets evaluation among $CD8^+$ T cells. The shaded region indicates the normal reference ranges.

CD4/CD8 ratios compared with B-ALL and DLBCL. However, the observed transduction efficiency, fold expansion, and ratio were comparable with earlier studies using a similar approach.[8,15] Interestingly, the transduction efficiency was not associated with the *in vivo* expansion, where the samples with the lowest transduction (VELCART 04 and 09) also showed an increase in the *in vivo* expansion and persistence of CAR-T cells. However, the sample with the lowest expansion during manufacturing (VELCART 03) also had a marginal expansion *in vivo*.

The T cell fitness in the starting material plays an important role in the CAR-T cell outcome; numerous studies consistently identify the proportion of naive and central memory T cells associated with CAR-T cell proliferation, efficacy, and persistence post-infusion.[18–20] The chronic stimulation by lymphoma results in T cell exhaustion driven by transcription factors such as *TOX*, *EOMES*, *BATF*, and *IRF4*, which is further affected by the immunosuppressive microenvironment and more lines of therapy.[21] In our cohort, the B-ALL patients had a significantly higher proportion of naive T cells than the DLBCL patients, who tended to have a higher percentage of effector T cells before the start of manufacturing.

In contrast, the final CAR-T cell product had increased central memory T cells across the subtypes and disease phenotypes. While effector T cells are known to have significant cytotoxic activity, central memory has superior persistence and is associated with favorable clinical responses.[20,22,23] These memory T cells were known to persist longer with anti-tumor activity and long-term efficacy.[24,25] In this study, the B-ALL samples had central memory T cells with a median of 76% (range, 60%–88%) among CD4 and 66% (range, 21%–80%) among CD8 compared with the DLBCL 74% (range, 54%–79%) in CD4 and 41% (range, 20%–67%) in CD8. The increase in the central memory T cells in the product persisted on day 14 of the post-infusion sample, after which a transitory expansion of effector memory T cells was observed, as in previous reports.[26] The manufactured CAR-T cells express low levels of exhaustion or inhibitory checkpoint molecules like PD-1, TIM-3, and LAG-3 confirming less susceptibility for inhibitory signals from tumor cells and validating their non-exhausted and high functional phenotype. These CAR-T cells could lyse $CD19^+$ cells specifically and secrete IFN-γ, GM-CSF, IL-2, and TNF-α in 20 to 30-fold higher levels upon contact with target cells, establishing specificity.

Consistent with previous studies,[8] this phase 1 study employed a dose escalation strategy infusing patients with 0.5×10^6 to 2×10^6 CAR-T cells/kg. While no dose-limiting non-hematological toxicity was observed, based on previous reports with this vector and our own experience, we propose that a dose of 2×10^6 cells/kg would be adequate for safety, tolerability, and efficacy for future studies assessing response and long-term outcomes. These CAR-T cells were

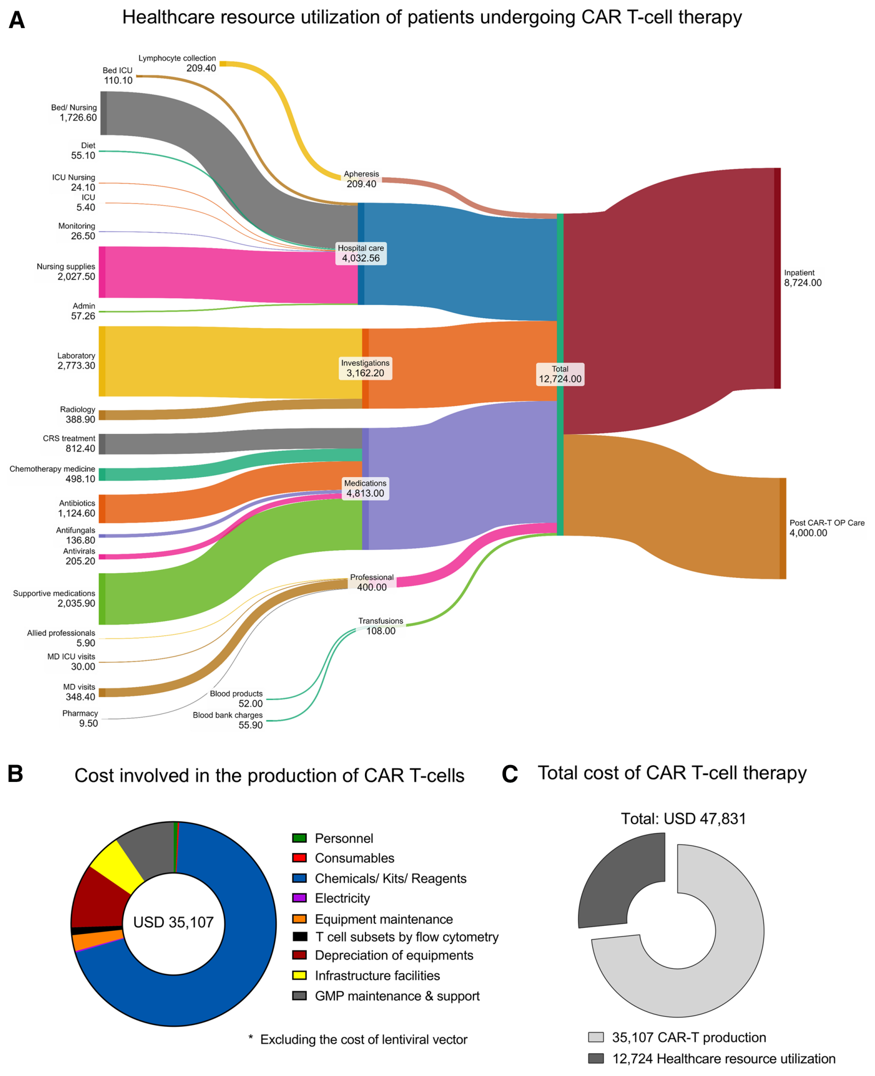

Figure 6. HRU and total cost of CAR-T cell therapy
(A) Sankey diagram illustrating the cost of health care resources during the entire treatment of CAR-T cell therapy as inpatient and post-CAR-T cell therapy outpatient care. The cost demonstrates the mean value of 10 patients involved in this study. The width of each flow represents the proportional quantity of the cost. The cost was represented as U.S. dollars. One dollar was calculated as 84 Indian rupees (based on the conversion during the analysis). (B) The doughnut plot depicts the percentage of cost involved in producing CAR-T cells with its major cost drivers, excluding the cost of lentiviral vector. (C) The sliced doughnut plot denotes the total cost of CAR-T cell therapy, which comprises the production cost and HRU of patients undergoing therapy.

highly efficient in inducing disease response in a cohort of heavily pre-treated patients, with CR achieved in 100% of ALL patients and 50% of DLBCL patients by 3 months. At a median follow-up of 15 months, 9 of the 10 patients remain without disease progression. Adverse events reported were CRS of grade 2 or higher in 2 out of 10 patients; none developed ICANS of any grade. Late hematological toxicity grade 2 or higher was seen only in one patient.

The CAR-T cell infusion follow-up assessment demonstrated B cell aplasia until day 90 and hypogammaglobulinemia beyond 1 year, indicating the long-lasting effect of CAR-T cells in patients. The lymphocyte subsets analysis revealed rapid recovery of T cells, NK cells, particularly $CD8^+$ effector T cells, establishing the cytotoxic activity against tumor cells.[17]

This study comprehensively evaluated a detailed cost breakdown using the actual cost from our accounting system. The hospital cost/HRU of patients undergoing CAR-T cell therapy, including in-patient and outpatient CAR T care, demonstrated a median cost of US $12,724. Adding this to our manufacturing cost of US$35,107, the total cost of this decentralized/POC manufactured CAR-T cell therapy is US$47,831, which is one-tenth of the cost of CAR-T cell products marketed in the United States.[27,28] However, the long-term disease response and detailed costing structures are yet to be evaluated.

To conclude, data from this study demonstrated that POC manufacturing of CAR-T cells with nine days of vein-to-vein time was feasible, safe, well tolerated, and efficacious using the CliniMACS Prodigy a fully automated closed system. The safety profile of this CAR T product was comparable with other CAR-T cell therapies, with higher CR rates across different doses in heavily pre-treated patients. It will be an effective treatment option for r/r B cell malignancies. This study was the first in the country to use the approach of the POC manufacturing strategy in an academic setting to reduce the cost burden involved in CAR-T cell therapy. We aim to increase access to CAR-T cell therapy in India by establishing POC manufacturing across multiple locations. Additionally, the same platform technology could be used at these centers to deliver lentiviral-based or other gene editing techniques for gene therapy for hemoglobinopathies, a relatively common genetic disorder in India, and possibly other rare genetic disorders.

METHODS

Study approvals

This study (VELCART) was approved by the Institutional Review Board (CMC IRB No: 12469), Institutional Biosafety Committee (CMC IBSC No: 12469) and the Review Committee on Genetic

Manipulation (RCGM No: BT/BS/17/159/2005-PID), Department of Biotechnology, India. The study was conducted after obtaining appropriate regulatory approvals [(CT-11/BD/02/2022), CT-06 and form-29 from the Central Drugs Standard Control Organization, Directorate General of Health Services, India]. The clinical trial was registered in the clinical trials registry—India CTRI/2022/10/046234. The CAR-T cells were manufactured at a good manufacturing process (GMP) facility (ISO class-7) at Christian Medical College, Vellore, India. All experiments and quality control assays were performed in-house.

Study details and toxicity monitoring

This study enrolled six r/r B-ALL and four r/r DLBCL between March 2023 to November 2023 with follow-up data to December 2024. This phase 1 study was designed with a 3 + 3 dose escalation strategy. The study was conducted according to the Declaration of Helsinki criteria, and all participants were enrolled after written informed consent.

The response was assessed by bone marrow MRD for B-ALL as per National Comprehensive Cancer Network guidelines[29] and positron emission tomography with computed tomography (PET CT) for DLBCL as per the Lugano classification.[30] The CRS and ICANS were graded and treated as per American Society for Transplantation and Cellular Therapy guidelines,[31] ICAHT was graded as per European Hematology Association/European Society for Blood and Bone Marrow Transplantation recommendations[32] and other toxicities were graded as per the National Cancer Institute's Common Terminology Criteria for Adverse Events CTCAE v5.[33] Patients with B-ALL were conceded to proceed with an allogeneic stem cell transplant after achieving MRD-negative status at the primary physician's discretion.

RESPONSE MONITORING

All patients had bone marrow aspiration/PET CT performed within 15–20 days before the lymphodepletion chemotherapy (single dose cyclophosphamide 40–60 mg/kg and fludarabine 30 mg/m^2 daily for 4 days). Post CAR-T cell infusion, bone marrow measurable residual disease (MRD) for B-ALL was performed on day 28 ± 2 days and at 3, 6, and 12 months after cell infusion, and a PET-CT for DLBCL was performed at 3, 6, and 12 months after cell infusion. MRD was assessed by multiparametric flow cytometry as described earlier[34] using BD FACSLyric (BD Biosciences). In addition, the CAR-T cell persistence was evaluated by flow cytometry on follow up days 3, 7, 14, 21, 28, 45, 60, and 90 post CAR-T cell infusion using MACSQuant10 flow cytometer (Miltenyi Biotec).

Manufacturing CAR-T cells using the CliniMACS Prodigy system

The CAR-T cells were manufactured using the CliniMACS Prodigy (Miltenyi Biotec) in our GMP facility, as described earlier.[12] Clinical-grade reagents from Miltenyi Biotec were used in this process, including CliniMACS Buffer, TexMACS Media, cytokines IL-7 and IL-15, CliniMACS CD4 and CD8 reagents, and T cell TransAct. The protocol and reagents were used according to the manufacturer's instructions. The TexMACS media was supplemented with IL-7 and IL-15 at the concentration of 12.5 ng/mL, and the CliniMACS PBS/EDTA buffer was supplemented with 0.5% human serum albumin (Intas Pharmaceuticals). After the T cells enrichment, ~1 × 10^8 T cells were utilized for cultivation. The cells were activated using TransAct, followed by transduction and culture expansion for nine days with automated media exchange every 3 days by protocol modification. The CAR-T cells were manufactured entirely in serum-free media.

Lentiviral vector

The second-generation CD19 CAR lentiviral vector (LTG1563, Miltenyi Biotec) with a single chain variable fragment FMC63-based targeting domain, 41BB co-stimulatory domain, CD8 hinge region, TNFRSF19 transmembrane domain, and CD3-zeta chain intracellular signaling domain was used at a multiplicity of infection of 16 for transduction. Data with this vector have been previously reported in two disparate sites, Russia and the United States.[8]

Cell lines

The cell lines NALM-6 and K562 were obtained from the American Type Culture Collection (ATCC). The cell lines were evaluated for mycoplasma contamination (Universal Mycoplasma Detection Kit, ATCC) and STR profiling (Geneprint10 kit, Promega) at regular intervals per laboratory standards. All the *in vitro* assays were carried out in TexMACS medium under serum-free conditions.

Flow cytometry

Flow cytometric characterization was done on fresh and unfixed cells. The cellular fractions before and after enrichment were analyzed using MACSQuant immune cell composition panel of antibodies CD45 VioBlue, CD4 VioGreen, CD3 FITC, CD16 PE, CD56 PE, CD19 PE-Vio770, CD14 APC, and CD8 APC-Vio770. The T cell transduction efficiency was determined using CD19 CAR detection reagent biotin-PE along with a panel of antibodies CD45 VioBlue, CD4 VioGreen, CD3 FITC, Biotin-PE, CD14 APC, and CD8 APC-Vio 770. We added 7AAD to these panels to discriminate against dead cells. T cell exhaustion and activation were evaluated using the following markers: CD366 APC, CD223 VioBlue, CD279 PE-Vio770, CD154 VioBlue, CD25 PE-Vio770, and CD137 APC. The T cell differentiation subsets were assessed using CD45RO APC, CD62L PE-Vio770 and CD45RA VioBlue, and standard T cell markers. The flow analysis of circulating CAR-T cells was done using CD19 CAR detection reagent. All the antibodies were obtained from Miltenyi Biotec, and the assay was performed according to the manufacturer's instructions using the MACSQuant 10 Flow cytometer (Miltenyi Biotech).

The CAR-T cell phenotype and immune subsets in the patient were done in DxFlex flow cytometer (Beckman Coulter) using the following antibodies: CD8 FITC, CD16 PE, CD56 PE, CD4 PerCPCy5.5, TCRγδ PE-Cy7, CD3 APC, CD45 APC-H7, CD27 BV421, CD45 RA BV510, 7-AAD (BD Biosciences), IgD-FITC, and IgM-PerCP-Cy5.5 (BioLegend), CD19 PC7, and CD64 ECD

(Beckman Coulter). The peripheral blood mononuclear cells were isolated and stained with a standardized antibody panel based on previous studies.[35] The data were analyzed using Kaluza analysis software v2.1 (Beckman Coulter) and Flow Jo v10.8.1 software (BD Biosciences).

Cytotoxicity assay

Target cells ($CD19^+$ NALM6 cell line) or non-target cells (CD19- K562 cell line) were stained with 0.5 μM concentration of CellTrace Violet dye (Thermo Fischer Scientific) according to the manufacturer's instructions. We co-cultured $\sim 1 \times 10^5$ cells with thawed and overnight rested CAR-T cells in different ratios from 1:1 to 1:10 in 96-well plates using TexMACS media. After overnight incubation, the cells were stained with viability dye 7AAD (BD Biosciences) and acquired in Navios flow cytometer (Beckman Coulter).

Antigen re-stimulation assay

To evaluate the antigen-specific expansion of CAR-T cells, 0.5×10^6 of CAR-T cells were co-cultured with an equal number of target cells ($CD19^+$ cells - NALM6), and the proliferation of CAR-T cells was assessed using CAR-detection reagent where the proliferation of tumor cells was evaluated using CD19 marker by flow cytometry. The cells were restimulated every 48 h with an equal number of target cells.

Cytokine analysis

We co-cultured $\sim 1 \times 10^6$ CAR-T cells with an equal number of target or non-target cells and incubated overnight in TexMACS media. After incubation, the levels of cytokines were estimated using the supernatant with respective controls. The cytokine analysis was performed using MACSplex cytokine 12 kit (Miltenyi Biotec) with a panel of cytokines GM-CSF, IFN-α, IFN-γ, IL-2, IL-4, IL-5, IL-6, IL-9, IL-10, IL-12p70, IL-17A, and TNF-α. The assay was performed using a MACSQuant flow cytometer (Miltenyi Biotec). Peripheral blood plasma was used to estimate the level of cytokines in patients' post CAR T infusion. The samples were diluted 1:10 with media or buffer following the manufacturer's instructions.

Proviral detection

The vector copy number TaqMan-based qPCR was done using proviral GAG DNA sequence and replication-competent lentivirus was determined using viral envelope sequence (VSVG). The DNA was extracted from CAR-T cells using a DNeasy blood and tissue kit (Qiagen), and qPCR was done in a C1000 Thermal Cycler (Bio-Rad).

Sterility assays

Mycoplasma was evaluated using a PCR-based assay utilizing a universal mycoplasma detection kit (ATCC). Endotoxin testing was done using an Endosafe PTS kit as per the manufacturer's protocol (Charles River Laboratories, Massachusetts, USA). Microbial cultures were assessed in accredited clinical service laboratories at CMC Vellore, India.

Cost analysis

Activity-based cost analysis was utilized from the perspective of the health care service provider. The cost data was collected from our hospital accounting system. The hospital length of stay and HRU were evaluated 7 days before CAR-T cell infusion and 60 days after infusion, including in-patient, outpatient, and other facility visits. The Sankey diagram was plotted using the sankeyMATIC tool.

Statistics

All data were analyzed using GraphPad Prism v8.0 (GraphPad Software, CA, USA). An unpaired two-tailed Student's t test was used to compare mean values between the two groups. Data points were represented in the graph as mean ± SEM. Statistical significance was given as *, **, *** for p values of less than 0.05, 0.01, and 0.001, respectively.

DATA AVAILABILITY

The authors confirm that the data supporting the findings of this study are present within the article and its supplemental information. All other data are available upon request to the corresponding author.

ACKNOWLEDGMENTS

This study is supported by an Indian Council of Medical Research (ICMR) grant (91/06/2020-TFGTR/BMS), New Delhi, India. We thank Miltenyi Biotech, Germany, for providing the lentiviral vector for this study. We also thank Cytocare Technologies, India, for support in the manufacturing process. We thank Mr. Christopher Benjamin, Ms. Nikath Jabeen, Mr. Muralish Eswar, and Mrs. Shruthi Pichandi for their help with sample and data acquisition. We also acknowledge technicians of the flow cytometry laboratory for their contribution to the T cell subsets and MRD assessments. We thank the doctors, nurses, and allied health personnel for the excellent care provided to these patients.

AUTHOR CONTRIBUTIONS

H.K.P. and V.M. conceived and designed the study. H.K.P., A.K.A., U.K., M.Y., A.V., S.P. K., R.N.R., M.S., A.R., P.V.D., S.S., A.K., A.A., B.G., and V.M. performed research, clinical data accrual, and reviewed data. A.K.A. performed the flow cytometric clustering analysis. A.B. performed the cost analysis. P.D., D.S., and L.W. provided critical guidance and support for the study. H.K.P. and V.M. drafted the article. All authors read and approved the final version of the manuscript.

DECLARATION OF INTERESTS

The authors declare no competing interests.

SUPPLEMENTAL INFORMATION

Supplemental information can be found online at https://doi.org/10.1016/j.omton.2025.200977.

REFERENCES

1. Kochenderfer, J.N., Dudley, M.E., Feldman, S.A., Wilson, W.H., Spaner, D.E., Maric, I., Stetler-Stevenson, M., Phan, G.Q., Hughes, M.S., Sherry, R.M., et al. (2012). B-cell depletion and remissions of malignancy along with cytokine-associated toxicity in a clinical trial of anti-CD19 chimeric-antigen-receptor-transduced T cells. Blood *119*, 2709–2720.

2. Maude, S.L., Laetsch, T.W., Buechner, J., Rives, S., Boyer, M., Bittencourt, H., Bader, P., Verneris, M.R., Stefanski, H.E., Myers, G.D., et al. (2018). Tisagenlecleucel in Children and Young Adults with B-Cell Lymphoblastic Leukemia. N. Engl. J. Med. *378*, 439–448.

3. Cappell, K.M., and Kochenderfer, J.N. (2023). Long-term outcomes following CAR T cell therapy: what we know so far. Nat. Rev. Clin. Oncol. *20*, 359–371.

4. Mitra, A., Barua, A., Huang, L., Ganguly, S., Feng, Q., and He, B. (2023). From bench to bedside: the history and progress of CAR T cell therapy. Front. Immunol. *14*, 1188049.

5. Hoffmann, M.S., Hunter, B.D., Cobb, P.W., Varela, J.C., and Munoz, J. (2023). Overcoming Barriers to Referral for Chimeric Antigen Receptor T Cell Therapy in Patients with Relapsed/Refractory Diffuse Large B Cell Lymphoma. Transplant. Cell. Ther. *29*, 440–448.

6. Ramakrishnan, S., Kumar, J., Datta, S.S., Radhakrishnan, V., Nair, R., and Chandy, M. (2022). Should we adopt an automated de-centralized model of chimeric antigen receptor- T cells manufacturing for low-and middle-income countries? A real world perspective. Front. Oncol. *12*, 1062296.

7. Mikhael, J., Fowler, J., and Shah, N. (2022). Chimeric Antigen Receptor T-Cell Therapies: Barriers and Solutions to Access. JCO Oncol. Pract. *18*, 800–807.

8. Maschan, M., Caimi, P.F., Reese-Koc, J., Sanchez, G.P., Sharma, A.A., Molostova, O., Shelikhova, L., Pershin, D., Stepanov, A., Muzalevskii, Y., et al. (2021). Multiple site place-of-care manufactured anti-CD19 CAR-T cells induce high remission rates in B-cell malignancy patients. Nat. Commun. *12*, 7200.

9. Kedmi, M., Shouval, R., Fried, S., Bomze, D., Fein, J., Cohen, Z., Danilesko, I., Shem-Tov, N., Yerushalmi, R., Jacoby, E., et al. (2022). Point-of-care anti-CD19 CAR T-cells for treatment of relapsed and refractory aggressive B-cell lymphoma. Transplant. Cell. Ther. *28*, 251–257.

10. Cliff, E.R.S., Kelkar, A.H., Russler-Germain, D.A., Tessema, F.A., Raymakers, A.J.N., Feldman, W.B., and Kesselheim, A.S. (2023). High Cost of Chimeric Antigen Receptor T-Cells: Challenges and Solutions. In American Society of Clinical Oncology Educational Book (ASCO publications), pp. e397912.

11. Shah, N.N., Zhu, F., Schneider, D., Krueger, W., Worden, A., Longo, W.L., Hamadani, M., Fenske, T.S., Dropulic, B., Orentas, R.J., et al. (2019). Fresh Versus Cryopreserved/Thawed Bispecific Anti-CD19/CD20 CAR-T Cells for Relapsed, Refractory Non-Hodgkin Lymphoma. Blood *134*, 4465.

12. Palani, H.K., Arunachalam, A.K., Yasar, M., Venkatraman, A., Kulkarni, U., Lionel, S.A., Selvarajan, S., Korula, A., Abraham, A., George, B., et al. (2023). Decentralized manufacturing of anti CD19 CAR-T cells using CliniMACS Prodigy®: real-world experience and cost analysis in India. Bone Marrow Transplant. *58*, 160–167.

13. Hernandez, I., Prasad, V., and Gellad, W.F. (2018). Total Costs of Chimeric Antigen Receptor T-Cell Immunotherapy. JAMA Oncol. *4*, 994–996.

14. Jagannath, S., Joseph, N., Crivera, C., Kharat, A., Jackson, C.C., Valluri, S., Cost, P., Phelps, H., Slowik, R., Klein, T., et al. (2023). Component Costs of CAR-T Therapy in Addition to Treatment Acquisition Costs in Patients with Multiple Myeloma. Oncol. Ther. *11*, 263–275.

15. Jackson, Z., Roe, A., Sharma, A.A., Lopes, F.B.T.P., Talla, A., Kleinsorge-Block, S., Zamborsky, K., Schiavone, J., Manjappa, S., Schauner, R., et al. (2020). Automated Manufacture of Autologous CD19 CAR-T Cells for Treatment of Non-Hodgkin Lymphoma. Front. Immunol. *11*, 1941.

16. Peinelt, A., Bremm, M., Kreyenberg, H., Cappel, C., Banisharif-Dehkordi, J., Erben, S., Rettinger, E., Jarisch, A., Meisel, R., Schlegel, P.-G., et al. (2022). Monitoring of Circulating CAR T Cells: Validation of a Flow Cytometric Assay, Cellular Kinetics, and Phenotype Analysis Following Tisagenlecleucel. Front. Immunol. *13*, 830773.

17. Ding, L., Cui, J., Hu, Y., Xu, H., Zhang, Y., Liu, S., Wang, K., Guo, Z., Chang, A., and Huang, H. (2018). Changes of T Lymphocyte Subsets after CAR-T Cell Therapy and Its Clinical Significance. Blood *132*, 1423.

18. Arunachalam, A.K., Grégoire, C., Coutinho de Oliveira, B., and Melenhorst, J.J. (2024). Advancing CAR T-cell therapies: Preclinical insights and clinical translation for hematological malignancies. Blood Rev. *68*, 101241.

19. Chen, G.M., Chen, C., Das, R.K., Gao, P., Chen, C.H., Bandyopadhyay, S., Ding, Y. Y., Uzun, Y., Yu, W., Zhu, Q., et al. (2021). Integrative Bulk and Single-Cell Profiling of Premanufacture T-cell Populations Reveals Factors Mediating Long-Term Persistence of CAR T-cell Therapy. Cancer Discov. *11*, 2186–2199.

20. Xu, Y., Zhang, M., Ramos, C.A., Durett, A., Liu, E., Dakhova, O., Liu, H., Creighton, C.J., Gee, A.P., Heslop, H.E., et al. (2014). Closely related T-memory stem cells correlate with in vivo expansion of CAR.CD19-T cells and are preserved by IL-7 and IL-15. Blood *123*, 3750–3759.

21. Zebley, C.C., and Youngblood, B. (2022). Mechanisms of T cell exhaustion guiding next-generation immunotherapy. Trends Cancer *8*, 726–734.

22. Berger, C., Jensen, M.C., Lansdorp, P.M., Gough, M., Elliott, C., and Riddell, S.R. (2008). Adoptive transfer of effector CD8+ T cells derived from central memory cells establishes persistent T cell memory in primates. J. Clin. Investig. *118*, 294–305.

23. Robbins, P.F., Dudley, M.E., Wunderlich, J., El-Gamil, M., Li, Y.F., Zhou, J., Huang, J., Powell, D.J., Jr., and Rosenberg, S.A. (2004). Cutting Edge: Persistence of Transferred Lymphocyte Clonotypes Correlates with Cancer Regression in Patients Receiving Cell Transfer Therapy. J. Immunol. *173*, 7125–7130.

24. López-Cantillo, G., Urueña, C., Camacho, B.A., and Ramírez-Segura, C. (2022). CAR-T Cell Performance: How to Improve Their Persistence? Front. Immunol. *13*, 878209.

25. Fraietta, J.A., Lacey, S.F., Orlando, E.J., Pruteanu-Malinici, I., Gohil, M., Lundh, S., Boesteanu, A.C., Wang, Y., O'Connor, R.S., Hwang, W.-T., et al. (2018). Determinants of response and resistance to CD19 chimeric antigen receptor (CAR) T cell therapy of chronic lymphocytic leukemia. Nat. Med. *24*, 563–571.

26. Blaeschke, F., Stenger, D., Kaeuferle, T., Willier, S., Lotfi, R., Kaiser, A.D., Assenmacher, M., Döring, M., Feucht, J., and Feuchtinger, T. (2018). Induction of a central memory and stem cell memory phenotype in functionally active CD4(+) and CD8(+) CAR T cells produced in an automated good manufacturing practice system for the treatment of CD19(+) acute lymphoblastic leukemia. Cancer Immunol. Immunother. *67*, 1053–1066.

27. Bach, P.B., Giralt, S.A., and Saltz, L.B. (2017). FDA Approval of Tisagenlecleucel: Promise and Complexities of a $475 000 Cancer Drug. JAMA *318*, 1861–1862.

28. Ran, T., Eichmüller, S.B., Schmidt, P., and Schlander, M. (2020). Cost of decentralized CAR T-cell production in an academic nonprofit setting. Int. J. Cancer *147*, 3438–3445.

29. Brown, P.A., Shah, B., Advani, A., Aoun, P., Boyer, M.W., Burke, P.W., DeAngelo, D. J., Dinner, S., Fathi, A.T., Gauthier, J., et al. (2021). Acute Lymphoblastic Leukemia, Version 2.2021, NCCN Clinical Practice Guidelines in Oncology. J. Natl. Compr. Canc. Netw. *19*, 1079–1109.

30. Cheson, B.D., Fisher, R.I., Barrington, S.F., Cavalli, F., Schwartz, L.H., Zucca, E., Lister, T.A., Alliance, Australasian Leukaemia and Lymphoma Group, Eastern Cooperative Oncology Group, European Mantle Cell Lymphoma Consortium, Italian Lymphoma Foundation, et al. (2014). Recommendations for initial evaluation, staging, and response assessment of Hodgkin and non-Hodgkin lymphoma: the Lugano classification. J. Clin. Oncol. *32*, 3059–3068.

31. Lee, D.W., Santomasso, B.D., Locke, F.L., Ghobadi, A., Turtle, C.J., Brudno, J.N., Maus, M.V., Park, J.H., Mead, E., Pavletic, S., et al. (2019). ASTCT Consensus Grading for Cytokine Release Syndrome and Neurologic Toxicity Associated with Immune Effector Cells. Biol. Blood Marrow Transplant. *25*, 625–638.

32. Rejeski, K., Subklewe, M., Aljurf, M., Bachy, E., Balduzzi, A., Barba, P., Bruno, B., Benjamin, R., Carrabba, M.G., Chabannon, C., et al. (2023). Immune effector cell–associated hematotoxicity: EHA/EBMT consensus grading and best practice recommendations. Blood *142*, 865–877.

33. (2017). Common Terminology Criteria for Adverse Events (CTCAE) V 5.0 (National Cancer Institute).

34. Arunachalam, A.K., Selvarajan, S., Mani, T., Janet, N.B., Maddali, M., Lionel, S.A., Kulkarni, U., Korula, A., Aboobacker, F.N., Abraham, A., et al. (2023). Clinical significance of end of induction measurable residual disease monitoring in B-cell acute lymphoblastic leukemia: A single center experience. Cytometry B Clin. Cytom. *104*, 440–452.

35. van der Burg, M., Kalina, T., Perez-Andres, M., Vlkova, M., Lopez-Granados, E., Blanco, E., Bonroy, C., Sousa, A.E., Kienzler, A.-K., Wentink, M., et al. (2019). The EuroFlow PID Orientation Tube for Flow Cytometric Diagnostic Screening of Primary Immunodeficiencies of the Lymphoid System. Front. Immunol. *10*, 246.

OMTON, Volume *33*

Supplemental information

Safety, efficacy and total cost of point-of-care manufactured anti-CD19 CAR-T cell therapy in India: VELCART trial

Hamenth Kumar Palani, Arun Kumar Arunachalam, Uday Kulkarni, Mohammed Yasar, Arvind Venkatraman, Swathy Palanikumar, Reeshma Nair Radhakrishnan, Majeela Solomon, Abirami Rajasekaran, Aniket Bankar, Phaneendra Venkateswara Rao Datari, Sushil Selvarajan, Anu Korula, Pradyot Dash, Dina Schneider, Louisa Wirthlin, Aby Abraham, Biju George, and Vikram Mathews

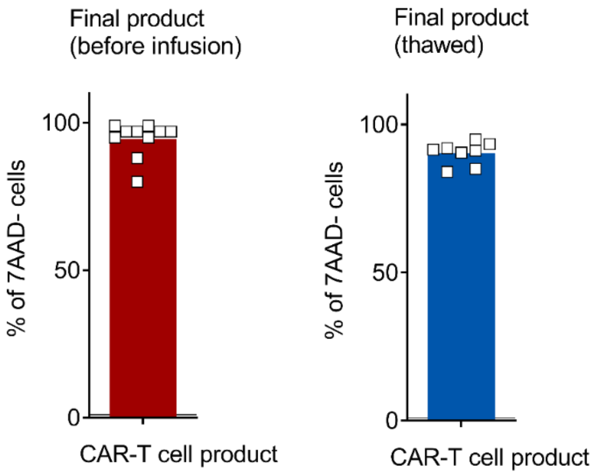

Figure S1. Viability analysis of cryopreserved CAR T-cell products thawed after six months. The viability was evaluated using 7AAD staining by flow cytometry after overnight resting with a TexMACS medium.

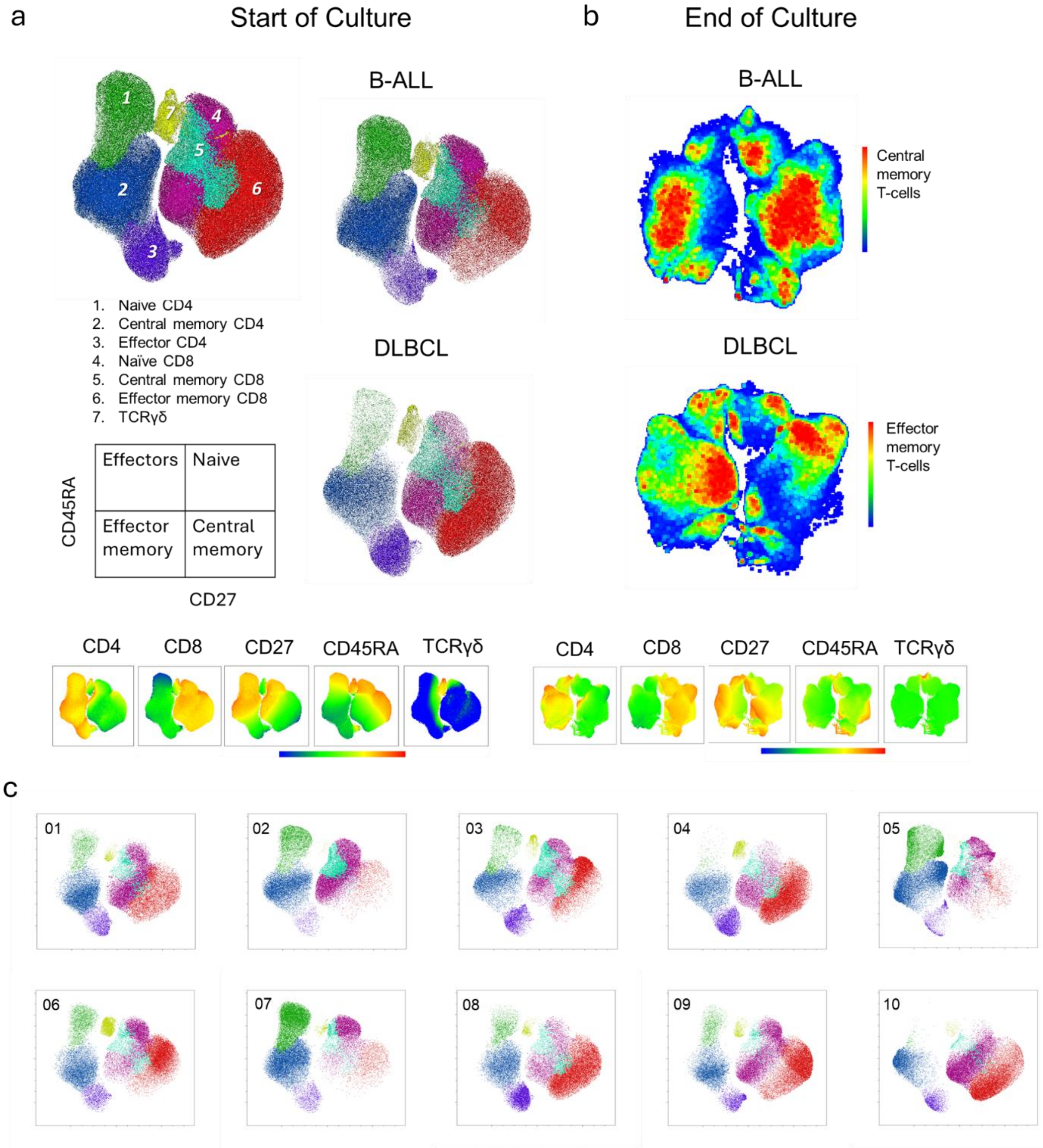

Figure S2. Identification and clustering of starting T-cells based on marker expression profiles. a) Clustering analysis of starting T-cells before culture. T-cells were identified with CD3 after sequentially excluding doublets, dead cells, CD45 negative events, B-cells, and monocytes. Unsupervised clustering via FLOWSOM, after dimensionality reduction through UMAP using FlowJo™ software. Multigraph color mapping demonstrates the expression of different markers.

b) Two-dimensional UMAP (uniform manifold approximation and projection) plot and the corresponding heatmap statistics showing the increased density of CD27 negative effector T-cells (CD4 & CD8) in the DLBCL samples and CD27 positive central memory T-cells (CD4 & CD8) in the B-ALL samples. c) The distribution of all clusters in the starting T-cells of all samples in the cohort.

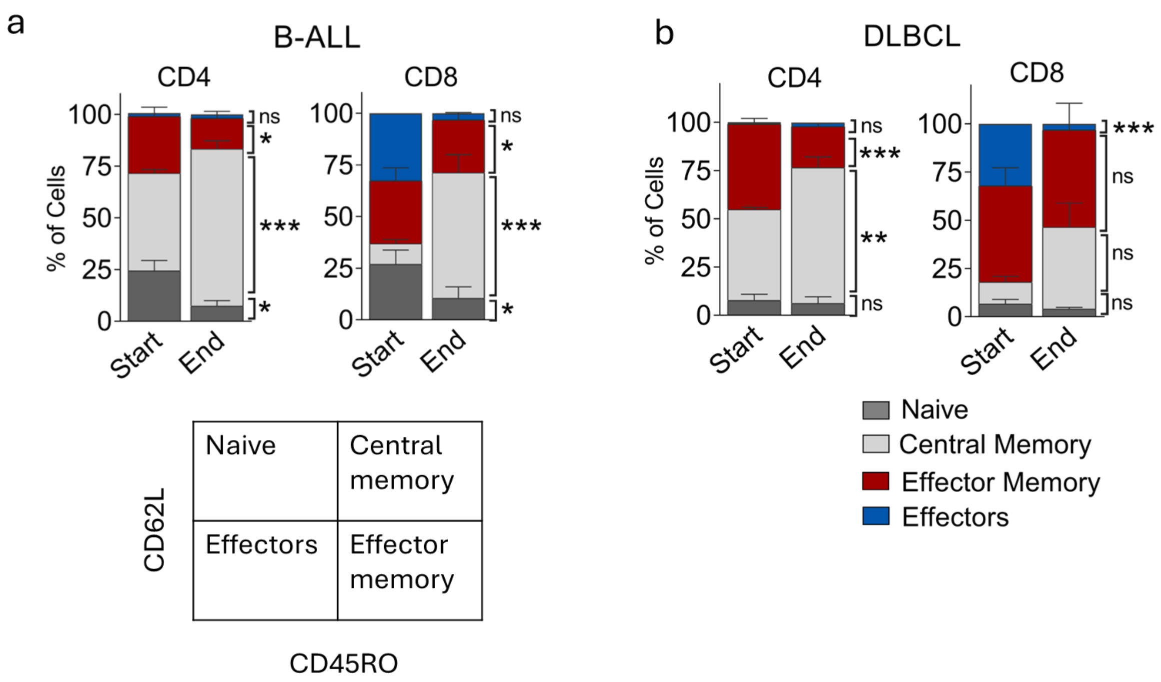

Figure S3. T-cell subset analysis of final CAR T-cell products comparing their starting T-cells. a) Percentage of cells in CD4 and CD8 subsets comparing the starting T-cell culture and final CAR T-cell products in B-ALL and DLBCL cohorts (b).

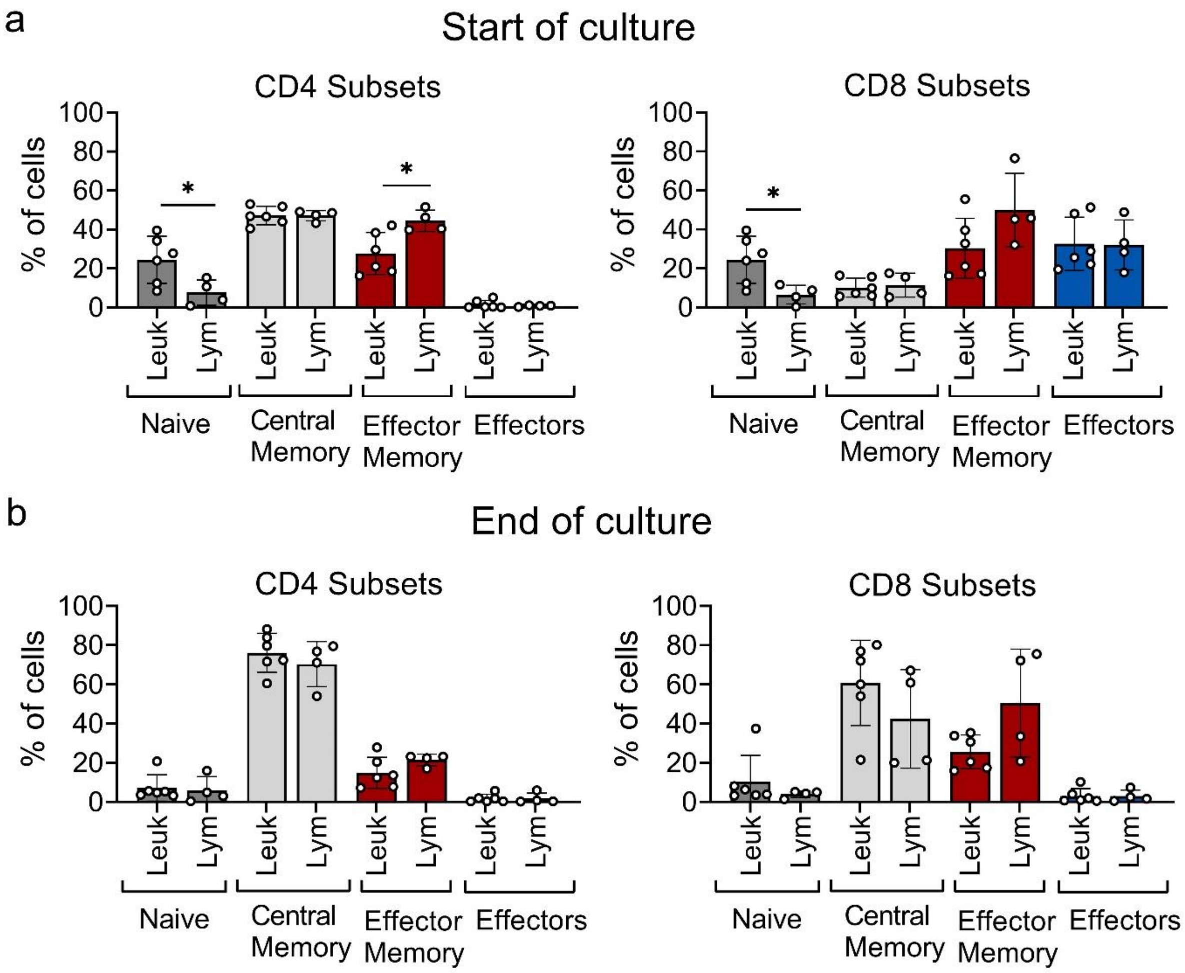

Figure S4. T-cell subset analysis of CAR T-cell products comparing leukemia and lymphoma. a) Immunophenotypic characterization of T-cell subsets comparing leukemia (B-ALL) and lymphoma (DLBCL) samples at the start of culture. b) Comparison of T-cell subsets at the end of culture. Each dot represents each sample n=8.

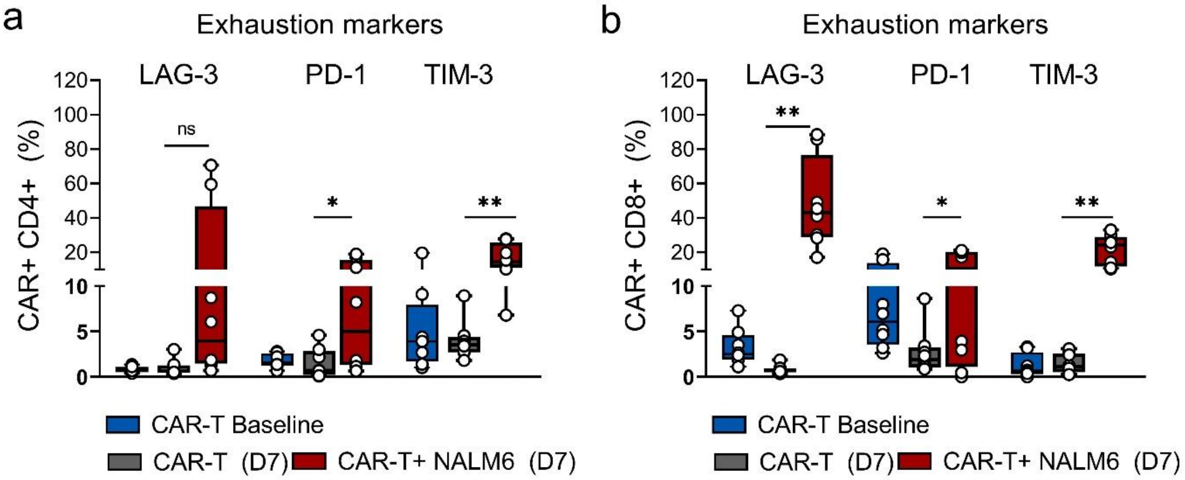

Figure S5. Expression of exhaustion marker on CAR T-cell restimulated with target cells. The expression of exhaustion makers of CAR T-cells after antigen-specific stimulation was done by an *in vitro* restimulation assay, where CAR T-cells were co-cultured with an equal number of target cells (NALM6) with intermittent stimulation for 6 days. a) Percentage of LAG-3, PD-1 and TIM-3 among CAR+ CD4+ cells. b) Percentage of markers among CAR+ CD8+ cells. Each dot represents each sample n=8.

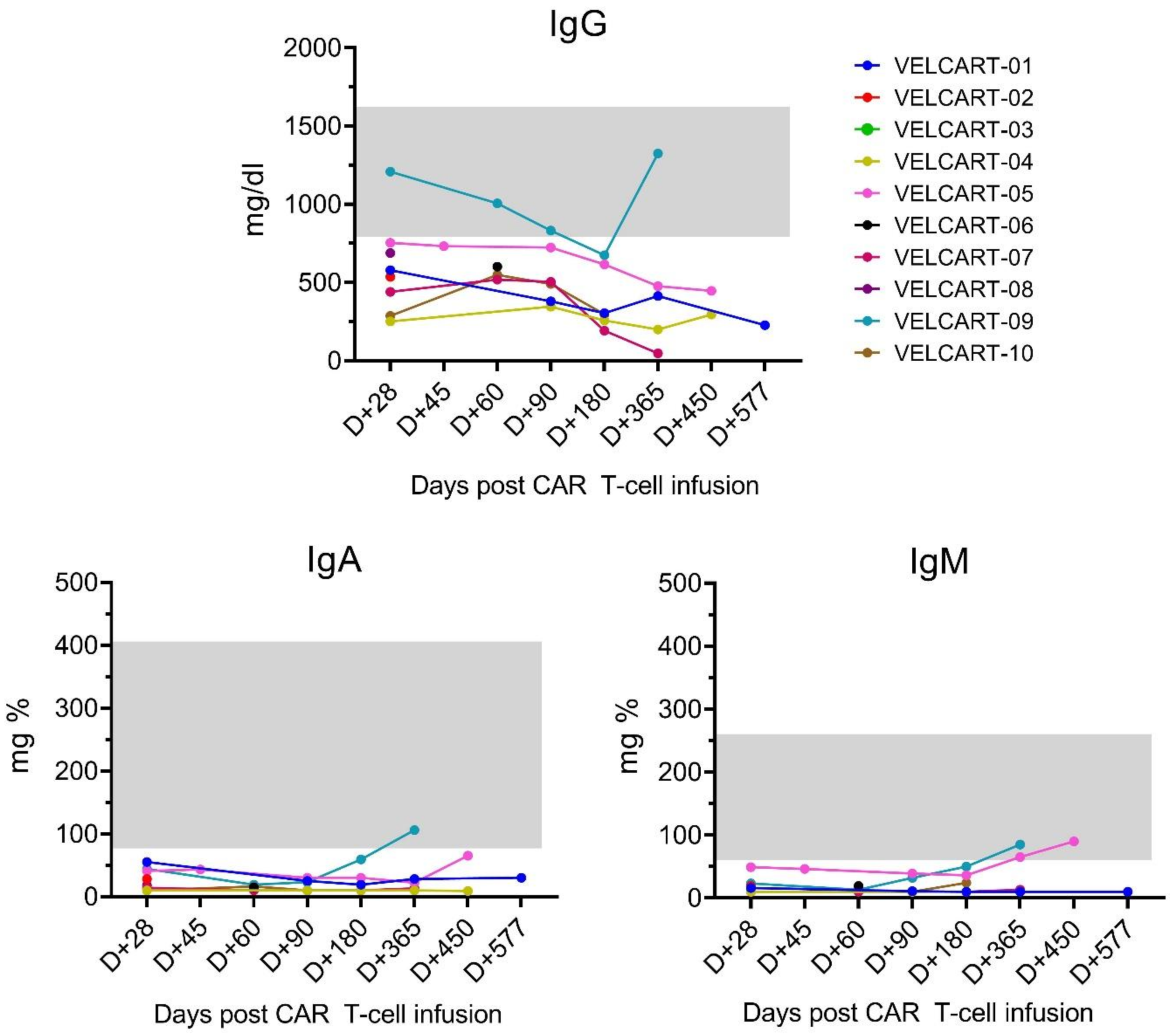

Figure S6. Serum immunoglobulin levels of patients post CAR T-cell infusion. Serum Immunoglobulin levels of IgG, IgA, and IgM in patients post CAR T-cell infusion. The shaded region indicates the normal reference ranges. The follow-up data was not available for VELCART 03 (died of progressive disease), VELCART 08 (withdrawn from study), and VELCART 02 and 06 (underwent HSCT).

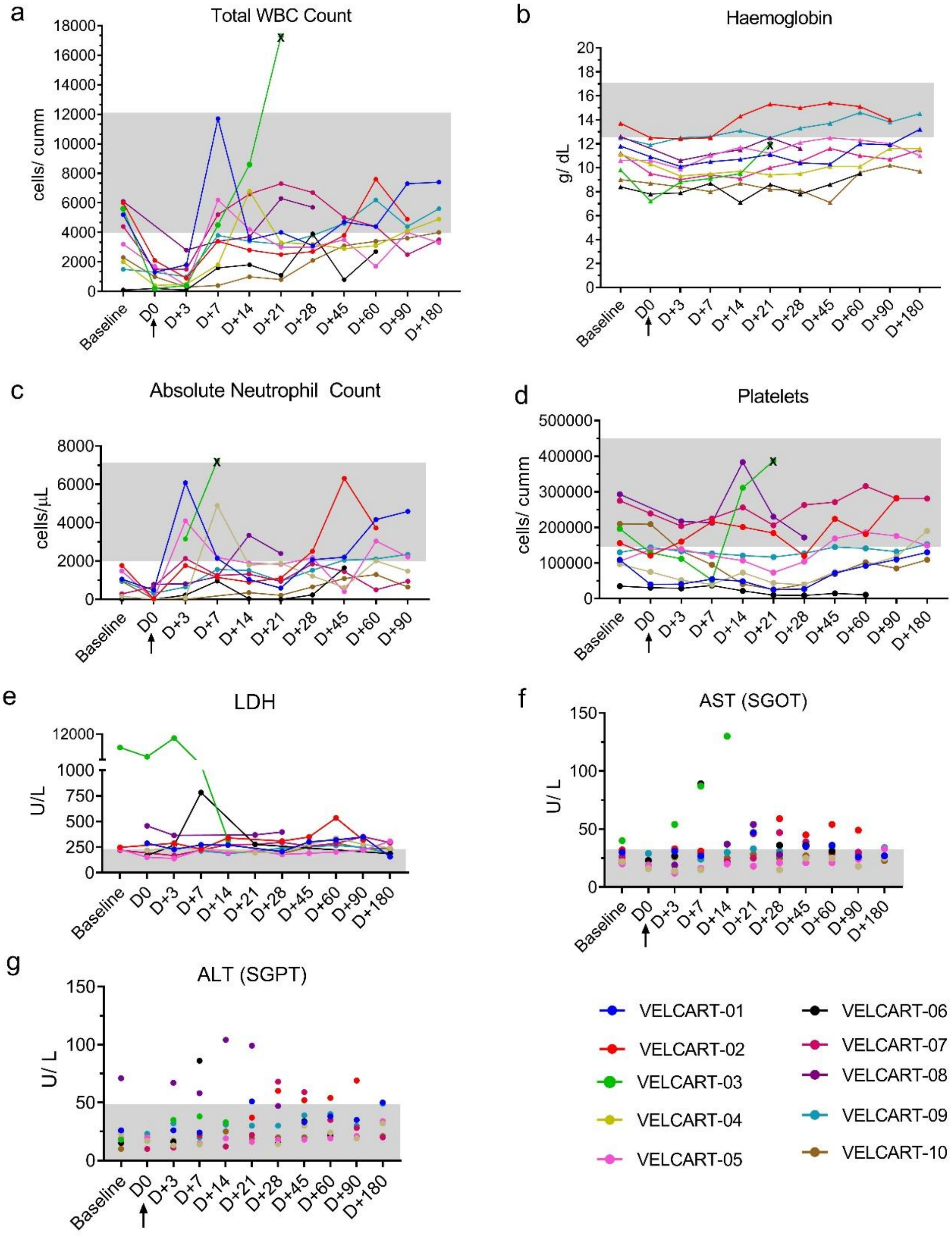

Figure S7. Patient demographics post CAR T-cell infusion. Peripheral blood assessments of cell count. LDH - Lactate Dehydrogenase, AST - aspartate aminotransferase, ALT - alanine aminotransferase. The shaded region indicates the normal reference ranges.

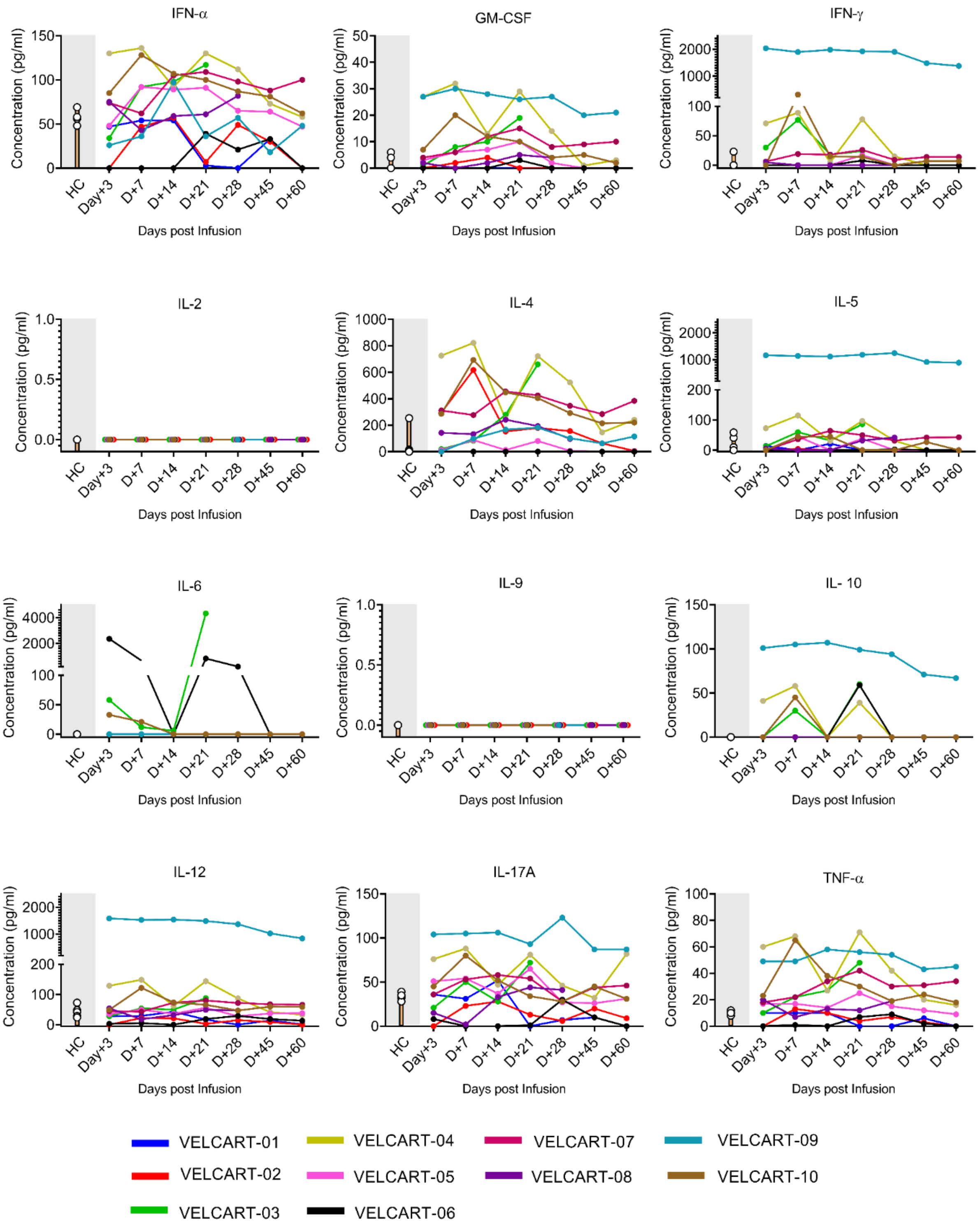

Figure S8. Plasma cytokines level assessments post CAR T-cell infusion. Cytokine levels of patients undergoing CAR T-cell therapy post-infusion in peripheral blood plasma. HC- Healthy controls (n=5).

Table S1. Patient's disease characteristics with treatment history

Patient	VELCART 01	VELCART 02	VELCART 03	VELCART 04	VELCART 05	VELCART 06	VELCART 07	VELCART 08	VELCART 09	VELCART 10
**Diagnosis**	Refractory B-ALL	Relapsed B-ALL	Relapsed refractory DLBCL	Relapsed refractory DLBCL	Refractory B-ALL	Relapsed refractory B-ALL	Relapsed B-ALL	Relapsed Refractory DLBCL	Refractory B-ALL	Primary refractory DLBCL
**Age & sex**	59 Male	20 Male	43 Male	59 Male	42 Male	29 Male	6 Male	48 Male	53 Male	57 Male
**No. of prior therapies & History**	4 LINES (Phase 1 Induction of GMALL Protocol - induction failure (bulk disease post-induction), three cycles of Mini HYPERCVAD + INO)	2 LINES: BFM 90 protocol - early medullary and CNS relapse - Mini Hyper CVAD+INO + TIT then one dose INO	2 LINES: 6 Cycles R-CHOP relapsed after 32 months - 5 Cycles RICE	6 LINES: RCHOP - relapsed after 29 months - RGCD, R-MINI BEAM, then Auto Tx done in PR f/b IFRT - then relapsed after 15 months - R-lenalidomide protocol	3 LINES: BFM 95 Adult ALL protocol phase 1 - induction failure (bulk disease post-induction), Phase 2 Induction, consolidation	3 LINES (BFM protocol - late medullary relapse - phase 1 induction - BM remission, testicular relapse - orchidectomy - MRD relapse - 2 cycles of INO)	3 LINES: (BFM protocol - early medullary relapse - BFM relapse protocol R1-R2-R1-VCR/ Bortezomib)	4 LINES: (R-CHOP - relapsed after 13 months - R-GDP, RICE, Auto Tx - relapsed after 8 months)	3 Lines: Adult ALL protocol: Received Phase-1, Received Phase-2, Inotuzumab x 3 doses	4 Lines: R-CHOP x 6 cycles, R-DHAP: 2 cycles, R-lenalidomide-Ibrutinib x 4 cycles, R-lenalidomide-ibrutinib-Ventoclax x 1 cycles, Polatuzumab, Bendamustine, Rituximab x 1 cycle
**Disease status at enrolment**	Residual disease	Residual disease	Progressive disease	Progressive disease	Residual disease	Bulk residual disease	Residual disease	Relapsed disease	Residual disease	Progressive disease

**MRD/ PET CT**	MRD: 0.17 %	MRD: 0.8 %	PET: Metabolically active findings: Small bowel, Jejunum, Ileum, Mesenteric nodes, left external Iliac nodes	PET: Metabolically active findings: Small bowel, Jejunum, Ileum, Mesenteric nodes, left external Iliac nodes, Left Inguinal nodes	MRD: 2.27 %	MRD: 28 %	MRD: 0.5 %	PET: Metabolic active findings: Sub-centric level 2 nodes, Left Para-aortic mass, Peri-portal node	MRD: 0.06 %	PET: Necrotic para-aortic nodes, Inguinal nodes, Abdominal wall, a superior component of epigastric region, antrum of the stomach, Lesion in Upper abdominal wall
**No prior lines of therapy (weeks)**	4	2	2	6	3	6	3	4	3	4
**Time from the last therapy (weeks)**	7	9	8	6	6	3	7	42	5	8

Table S2. CAR T-cell product release criteria assays

Details/ Assay	Method	VELCART 01	VELCART 02	VELCART 03	VELCART 04	VELCART 05	VELCART 06	VELCART 07	VELCART 08	VELCART 09	VELCART 10
**Diagnosis**	NA	Refractory B-ALL	Relapsed B-ALL	Relapsed refractory DLBCL	Relapsed refractory DLBCL	Refractory B-ALL	Relapsed refractory B-ALL	Relapsed B-ALL	Refractory DLBCL	Refractory B-ALL	Primary refractory DLBCL
**Age & sex**	NA	59 Male	20 Male	43 Male	59 Male	42 Male	29 Male	6 Male	48 Male	53 Male	57 Male
**No of days culture**	NA	9	9	9	9	9	9	9	9	9	9
**Viability**	Flow cytometry 7AAD	99 %	97 %	88 %	97 %	97 %	95 %	80 %	98 %	99 %	99 %
**Transduction efficiency**	Flow cytometry – CAR %	40 %	37 %	45 %	18 %	36 %	47 %	55 %	55 %	16 %	35 %
**Phenotype harvest**	Flow cytometry CD3 + cells	99 %	99 %	99 %	98 %	99 %	99 %	99 %	99 %	99 %	99 %
**Sterility**	Microbial culture	No growth	No growth	No growth	No growth	No growth	No growth	No growth	No growth	No growth	No growth
**Gram stain**	Grams's methods	Negative	Negative	Negative	Negative	Negative	Negative	Negative	Negative	Negative	Negative
**Endotoxin**	Endosafe PTS	<0.05 EU/ml	<0.05 EU/ml	<0.05 EU/ml	<0.05 EU/ml	<0.05 EU/ml	<0.05 EU/ml	<0.05 EU/ml	<0.05 EU/ml	<0.05 EU/ml	<0.05 EU/ml
**Mycoplasma**	PCR	Negative	Negative	Negative	Negative	Negative	Negative	Negative	Negative	Negative	Negative
**Vector copy number**	QPCR	2.0 copies/ cell	2.6 copies/ cell	1.8 copies/ cell	1.8 copies/ cell	1.4 copies/ cell	2.1 copies/ cell	2.0 copies/ cell	2.0 copies/ cell	2.0 copies/ cell	2.0 copies/ cell
**VSVG**	QPCR	UND	UND	UND	UND	UND	UND	UND	UND	UND	UND

Table S3. Sample process information

Sample details	Cell product volume (ml)	Concentration of WBC ($X10^6$/ml)	Total WBCs ($X10^9$)	Total labeled cells ($X10^9$)	Frequency of labeled cells (%)	Seeding count ($X10^6$)
VELCART 01	105	71.5	7.5	3.1	42.4	100
VELCART 02	100	100	10	7.2	72	
VELCART 03	100	45	4.5	1.4	33	
VELCART 04	180	31	5.5	3.5	62.4	
VELCART 05	190	111	21	13	61.9	
VELCART 06	200	19	3.8	2.9	76.2	
VELCART 07	140	33	4.6	1.7	37.2	
VELCART 08	180	130	23.4	8.9	38.4	
VELCART 09	150	72.5	10.8	3.7	34.5	
VELCART 10	189	47	8.8	3.9	44.5	

Table S4. Purity of apheresis sample and recovery post-enrichment of CD4+ and CD8+ cells

Sample details	% Among viable CD45+ cells			
	CD 4+ cells (%)		CD 8+ cells (%)	
	Pre-column purity	Post-column recovery	Pre-column purity	Post-column recovery
VELCART 01	14.6	34.8	14.7	34.3
VELCART 02	30.1	41.6	35.8	51
VELCART 03	11.3	33	17.4	44.5
VELCART 04	15.3	25.8	34	58.5
VELCART 05	40.3	64.4	13.7	27
VELCART 06	23.2	34.3	29.2	35
VELCART 07	22	70.5	7.2	21.5
VELCART 08	14.3	35.7	19.3	50.8
VELCART 09	9.1	27.6	17.2	60
VELCART 10	10	19.6	26	59.1

Table S5. Toxicities post CAR T-cell infusion comparing the dose levels

Sample details	Dose	CRS	ICANS	ICAHT	Hepatotoxicity	Nephro toxicity	Cardio toxicity
VELCART 01	0.5×10^6/ Kg	Grade 1	Nil	Grade 1	Nil	Nil	Nil
VELCART 02	0.5×10^6/ Kg	Grade 2	Nil	Grade 1	Grade 1	Nil	Nil
VELCART 03	0.5×10^6/ Kg	Grade 1	Nil	Grade 1	Grade 2	Nil	Nil
VELCART 04	1×10^6/ Kg	Grade 1	Nil	Grade 2	Nil	Nil	Nil
VELCART 05	1×10^6/ Kg	Grade 1	Nil	Grade 1	Nil	Nil	Nil
VELCART 06	1×10^6/ Kg	Grade 3	Nil	Grade 3	Nil	Nil	Nil
VELCART 07	2×10^6/ Kg	Grade 1	Nil	Grade 1	Grade 1	Nil	Nil
VELCART 08	2×10^6/ Kg	Grade 1	Nil	Grade 1	Nil	Nil	Nil
VELCART 09	2×10^6/ Kg	Grade 1	Nil	Grade 1	Nil	Nil	Nil
VELCART 10	2×10^6/ Kg	Grade 1	Nil	Grade 2	Nil	Nil	Nil

Supplemental Methods

Clustering analysis

Different T-cell subsets were identified and clustered by comparing the B-ALL and DLBCL samples based on their marker expression profiles. Naïve (CD27+ CD45RA+), Central memory (CD27+CD45RA-), Effector memory (CD27-CD45RA-), Terminally differentiated (CD27-CD45RA+). T-cells were identified with CD3 after sequentially excluding doublets, dead cells, CD45-negative events, B-cells, and monocytes. Unsupervised clustering via FLOWSOM, after dimensionality reduction through UMAP, was performed on 400,000 T-cells (200,000 events from leukemia and lymphoma samples) using FlowJo™ software (BD Biosciences).
